# Spectroscopic dataset of Hedione's derivatives gathered during process development

**DOI:** 10.1016/j.dib.2022.108801

**Published:** 2022-12-05

**Authors:** James S. Sharley, Guido Gambacorta, Ana María Collado Pérez, Estela Espinos Ferri, Amadeo Fernandez Miranda, Jorge Sanchez Quesada, Ian R. Baxendale

**Affiliations:** a Department of Chemistry, University of Durham, South Road, Durham DH1 3LE, UK; b International Flavours & Fragrances Inc., Avda Felipe Klein 2, Benicarló, Castellón 12580, Spain

**Keywords:** Hedione, Chlorination, Dehydrohedione, NMR, Fluorination, Accurate mass

## Abstract

The dataset of spectroscopic analysis performed on starting materials, intermediates, and products relating to the synthesis of Hedione are hereby presented. The data were acquired in Durham university during the period between October 2020 and September 2021 for the development of a preparative method to Dehydrohedione. The latter is a key intermediate for the synthesis of *cis-*Hedione, an important fragrance ingredient. Proton, Carbon-13, and Fluorine-19 Nuclear magnetic resonance of the compounds were recorded employing a Varian 600 MHz, and a Bruker Avance-400 instrument. The IR spectra were recorded in a Perkin Elmer Spectrum Two UATR Two FT-IR and the accurate mass employing a Waters QtoF premier as mass spectrometer.

**Specifications Table**

**Table:** 

Subject	Organic Chemistry
Specific subject area	Process Development, Nuclear magnetic resonance, Organic Chemistry
Type of data	Graph Figure
How the data were acquired	NMR spectras - Bruker Avance-400, Varian VNMRS-600. IR spectra - Perkin Elmer Spectrum Two UATR Two FT-IR Spectrometer. High resolution mass spectrometry - Waters QtoF Premier.
Data format	Analyzed csv and fid data figures and pdf format
Description of data collection	NMR spectra: The samples were prepared by dissolving 20 mg of sample in CDCl_3_ and then transferred in a NMR tube for the data acquisition. FT-IR spectra: The samples were acquired by placing the neat compound on the spectrometer. High resolution mass: The samples were prepared by dissolving on 1–2 mg of sample in a 1 mL of HPLC grade acetonitrile prior acquisition on the instrument.
Data source location	Institution: Department of Chemistry, Durham University City/Town/Region: Durham, DH1 3LE Country: UK. Latitude and longitude and GPS coordinates, for collected samples/data: 54.7680° N, 1.5702° W, 54.768151, -1.571072
Data accessibility	NMR, FT-IR and HR-MS raw data: Repository name: Mendeley data Direct URL to data: Gambacorta, Guido; Baxendale, Ian (2022), “Characterisation of Hedione's derivative compounds”, Mendeley Data, V3, doi: doi:10.17632/2tfvzrmhzs.3
Related research article	Baxendale, Ian R. and Sharley, James S. and Gambacorta, Guido and Collado Pérez, Ana María and Ferri, Estela Espinos and Miranda, Amadeo Fernandez and Fernández, Isabelle Fernández and Quesada, Jorge Sanchez, A Simple One-Pot Oxidation Protocol for the Synthesis of Dehydrohedione from Hedione. Tetrahedron 2022, 126, 133068. Available at https://doi.org/10.1016/j.tet.2022.133068

## Value of the Data

• These analyses provide deep insights in the characterization of the described chemical compounds.
• The data may be used for the development of a new synthetic strategies to Hedione and its derivative.
• Chemist in the flavors and fragrances industry and research areas can take advantage of the easily accessed information.

## 1 Objective

These dataset [1] was acquired during the development of a preparative method to *cis-*Hedione, a potent and widely employed fragrance ingredient. These data provide insights into how the authors characterized the chemical compounds obtained during the investigation [2], providing a smoother and clearer presentation to the reader. Furthermore, this spectroscopic dataset will add information to the flavors and fragrances community and value by way of insights on the mechanism behind the chemical transformation reported in the related article (Fig. 1, Fig. 2, Fig. 3, Fig. 4, Fig. 5, Fig. 6, Fig. 7, Fig. 8, Fig. 9, Fig. 10, Fig. 11, Fig. 12, Fig. 13, Fig. 14, Fig. 15, Fig. 16, Fig. 17, Fig. 18, Fig. 19, Fig. 20, Fig. 21, Fig. 22, Fig. 23, Fig. 24, Fig. 25, Fig. 26, Fig. 27, Fig. 28, Fig. 29, Fig. 30, Fig. 31, Fig. 32, Fig. 33, Fig. 34, Fig. 35, Fig. 36, Fig. 37, Fig. 38, Fig. 39).

**Fig. 1:**
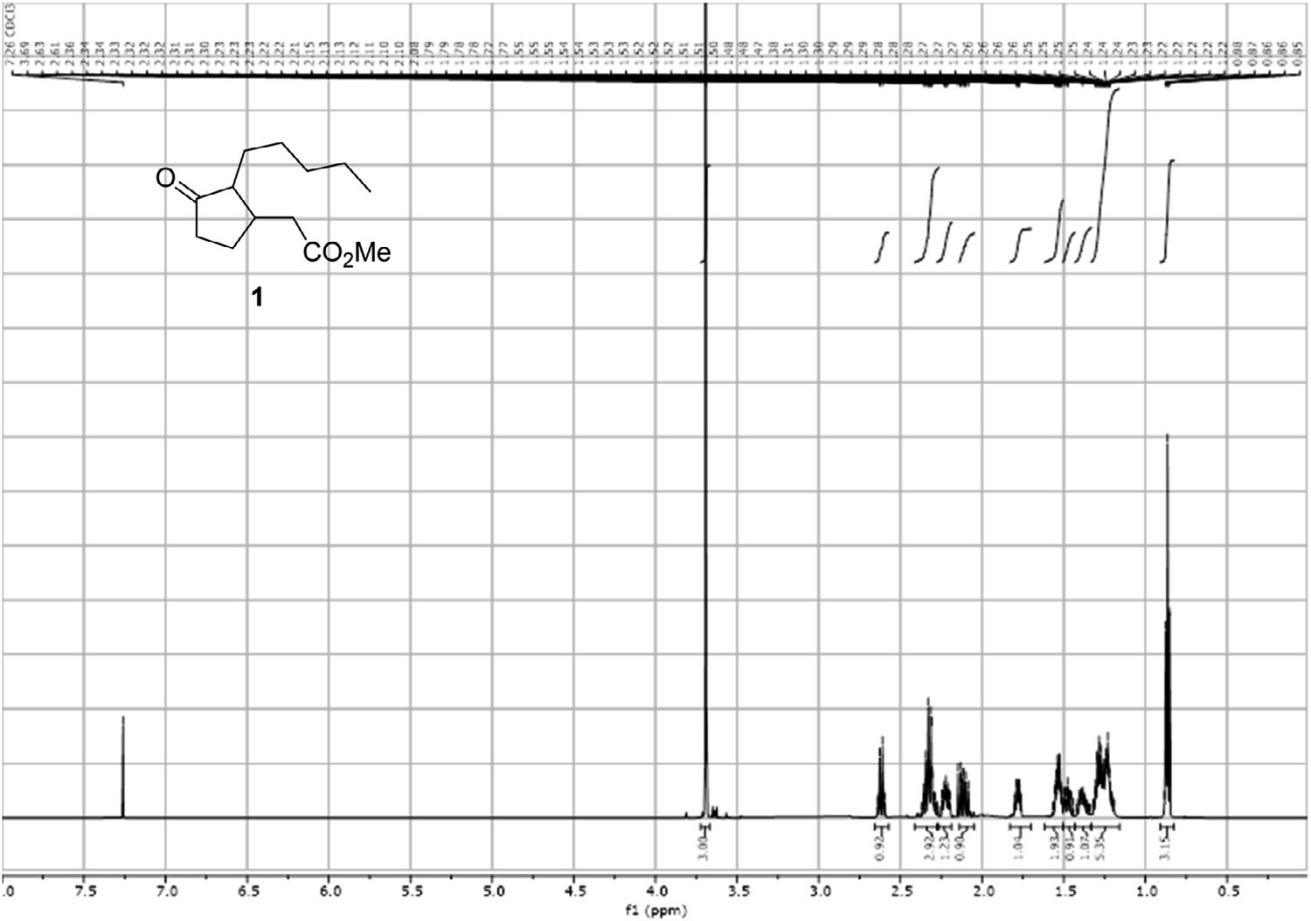
^1^H-NMR spectra for the compound **1**[2].

**Fig. 2:**
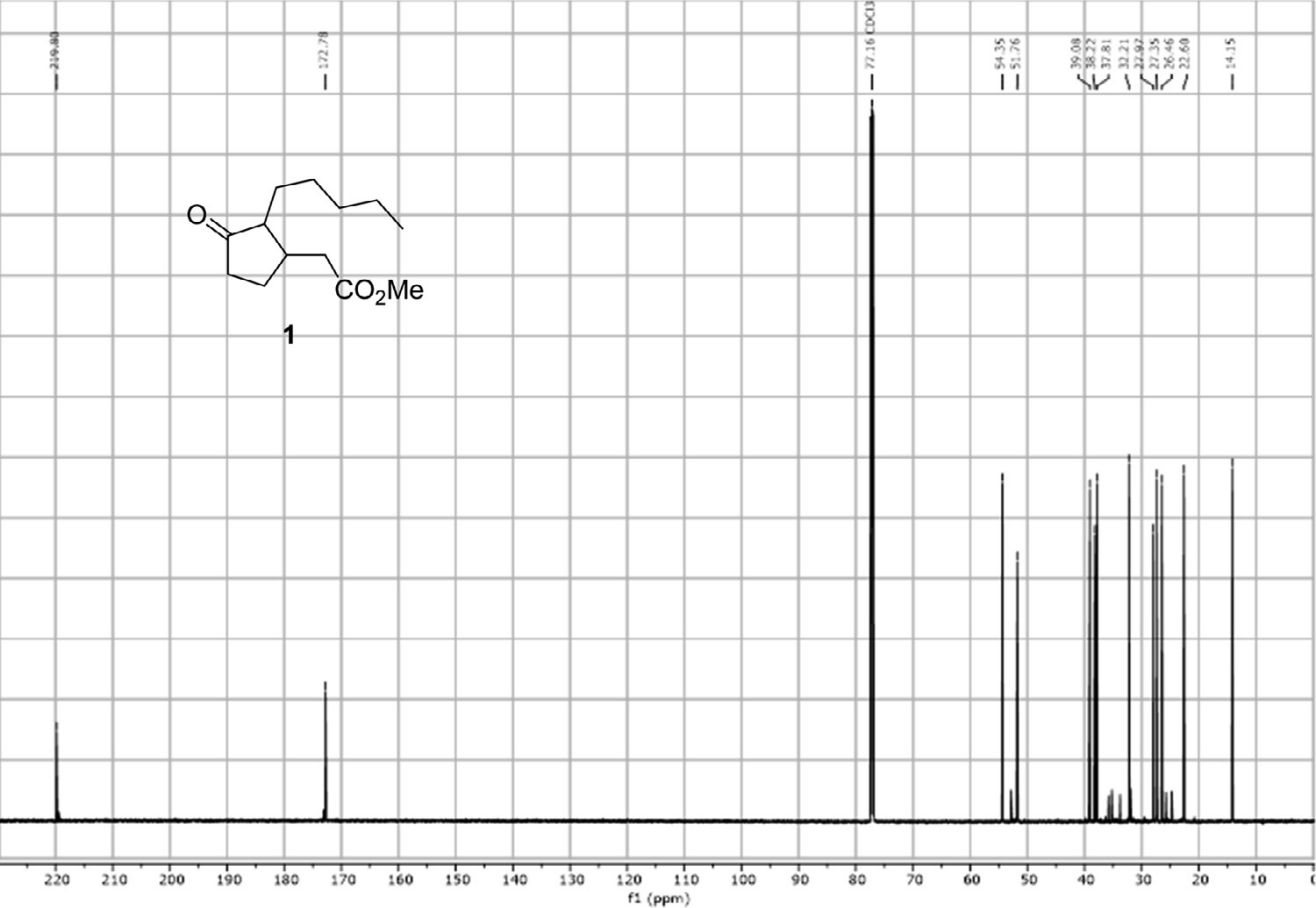
^13^C-NMR spectra of the compound **1**[2].

**Fig. 3:**
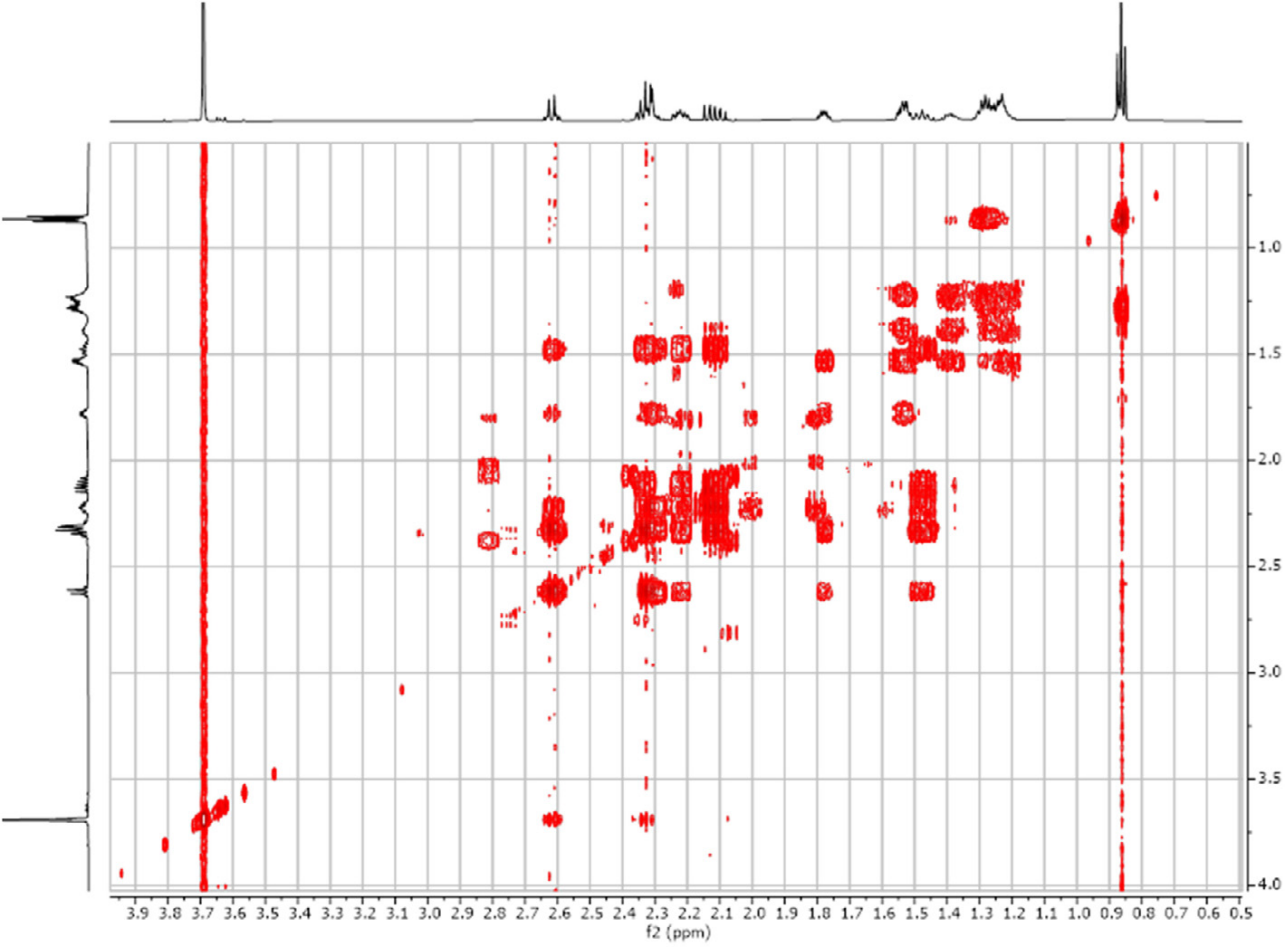
2-D ^1^H-^1^H COSY spectra for the compound **1**.

**Fig. 4:**
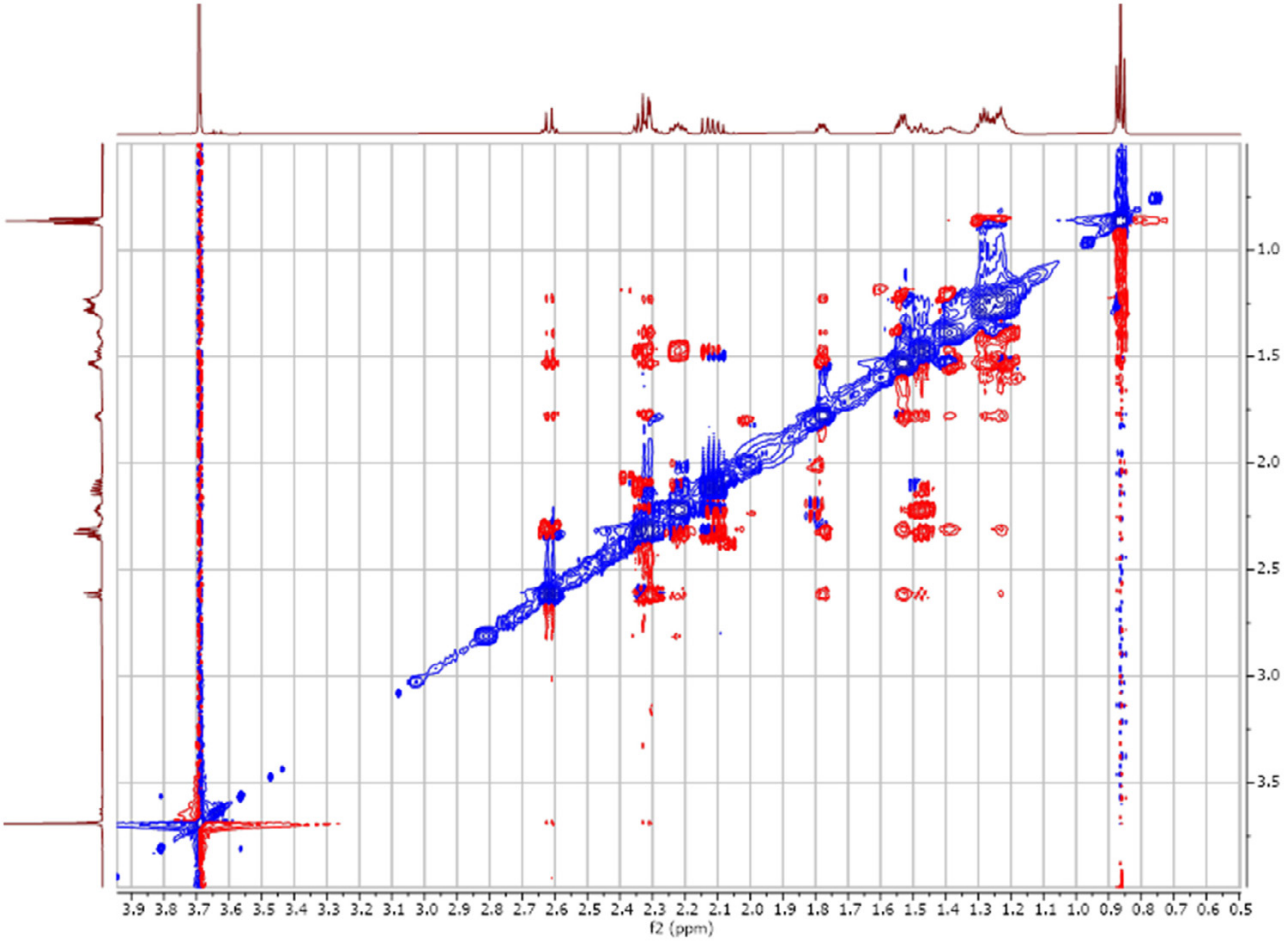
2-D ^1^H -^1^H NEOSY spectra for the compound **1**.

**Fig. 5:**
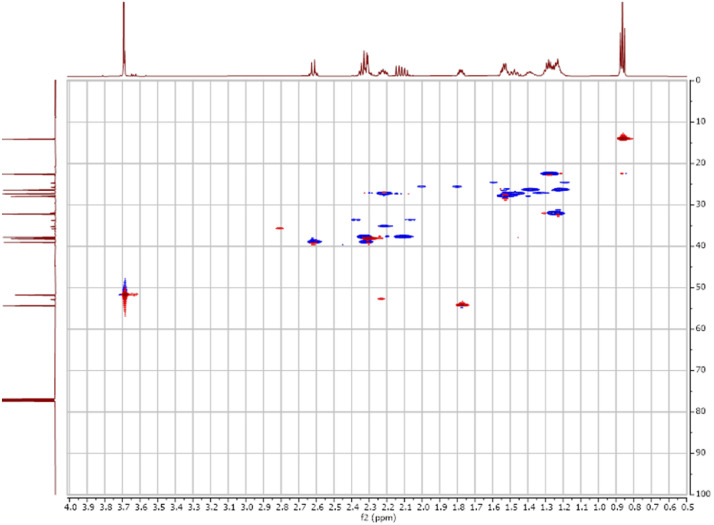
2-D ^1^H-^13^C HSQC spectra for the compound **1**.

**Fig. 6:**
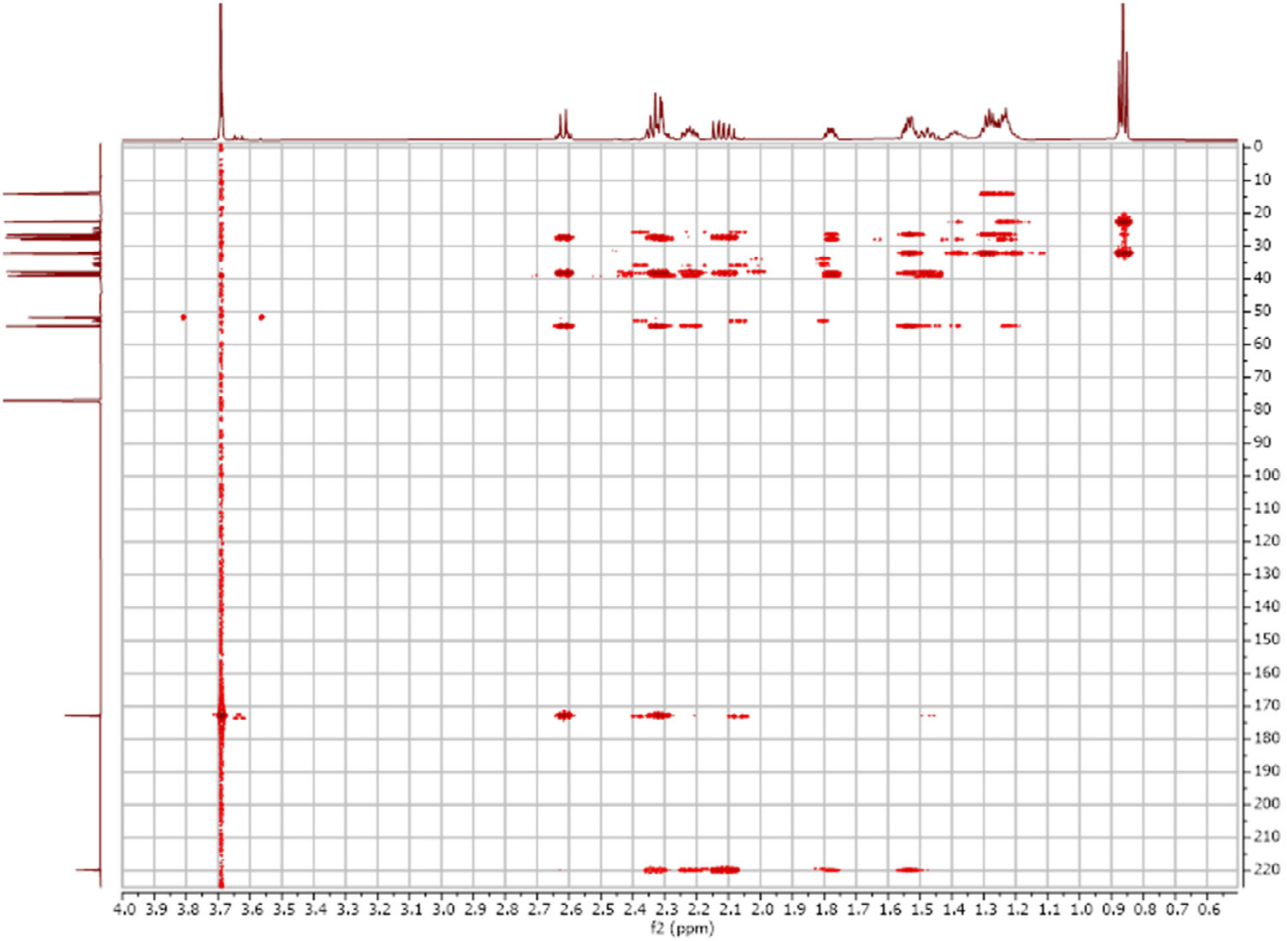
2-D ^1^H-^13^C HMBC spectra for the compound **1**.

**Fig. 7:**
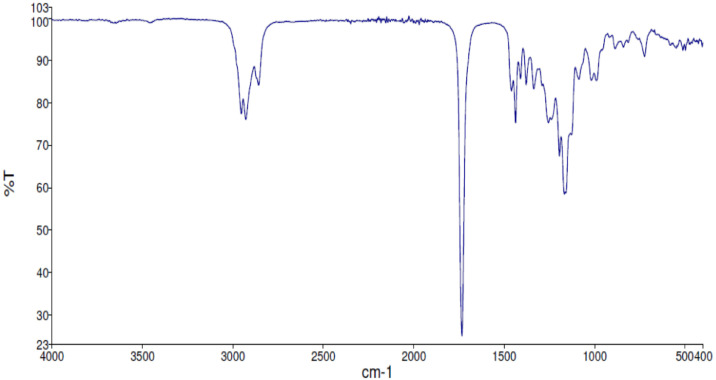
FT-IR spectra acquired for the compound **1**[2].

**Fig. 8:**
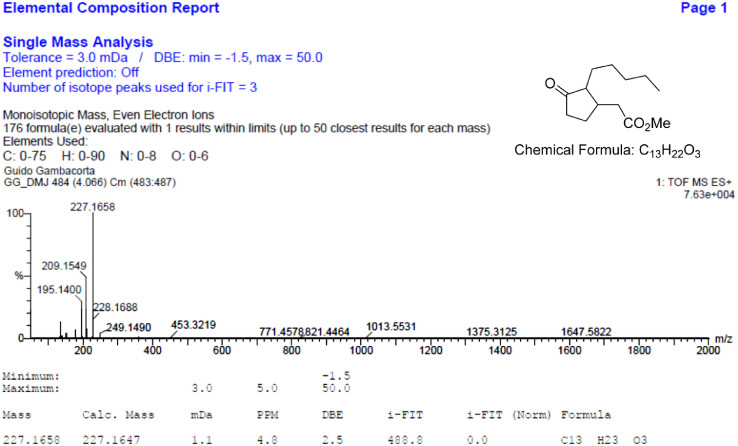
HR-MS report for the compound **1**[2].

**Fig. 9:**
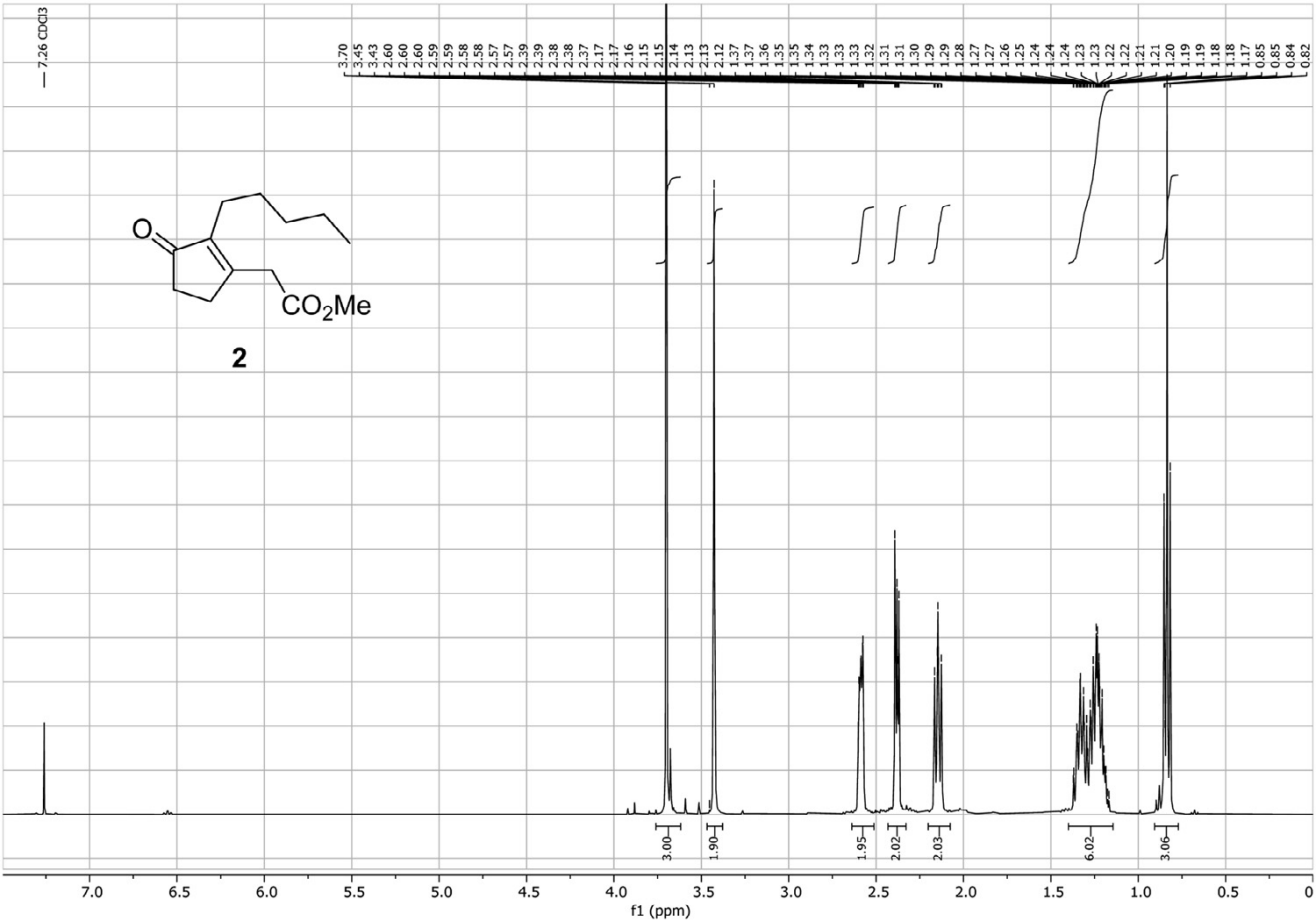
^1^H-NMR spectra for the compound **2**[2].

**Fig. 10:**
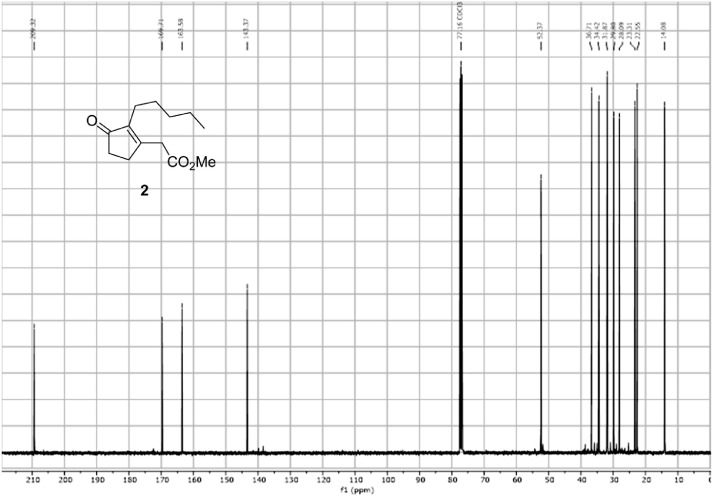
^13^C spectra for the compound **2**[2].

**Fig. 11:**
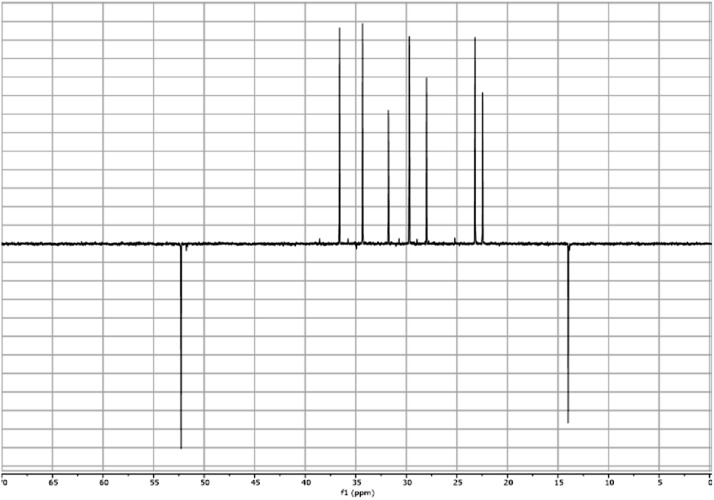
^13^C DEPT spectra for the compound **2**.

**Fig. 12:**
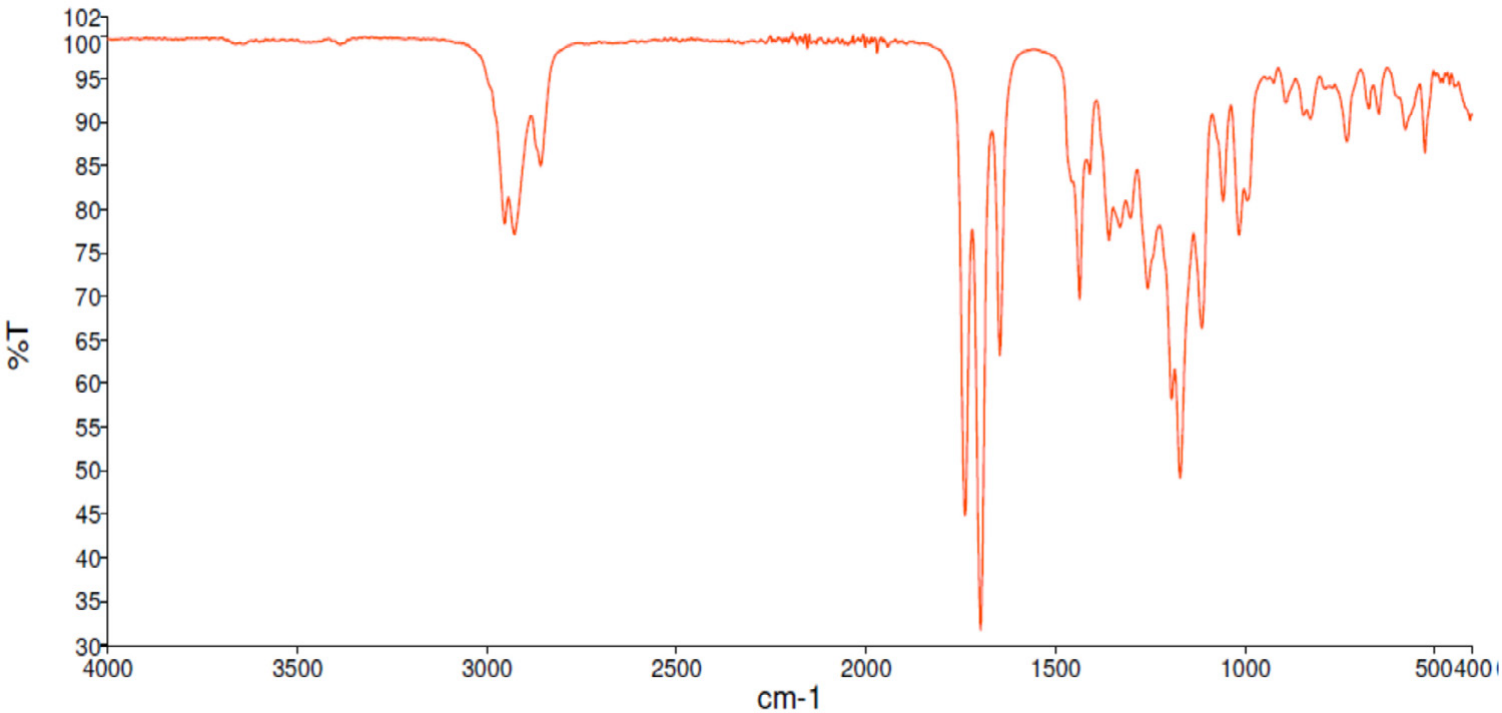
FT-IR spectra for the compound **2**[2].

**Fig. 13:**
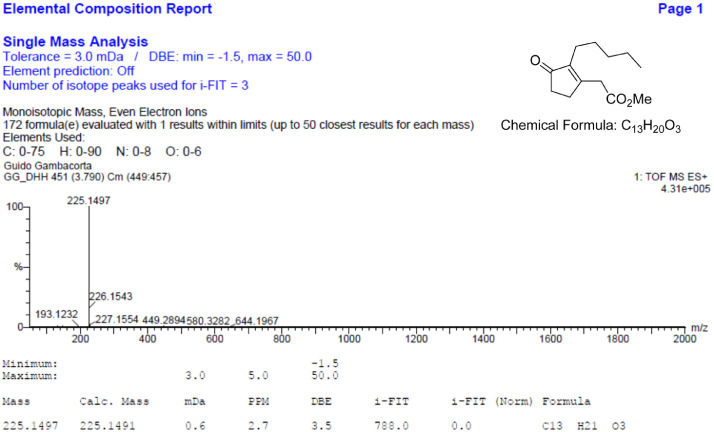
HR-MS report for the compound **2**[2].

**Fig. 14:**
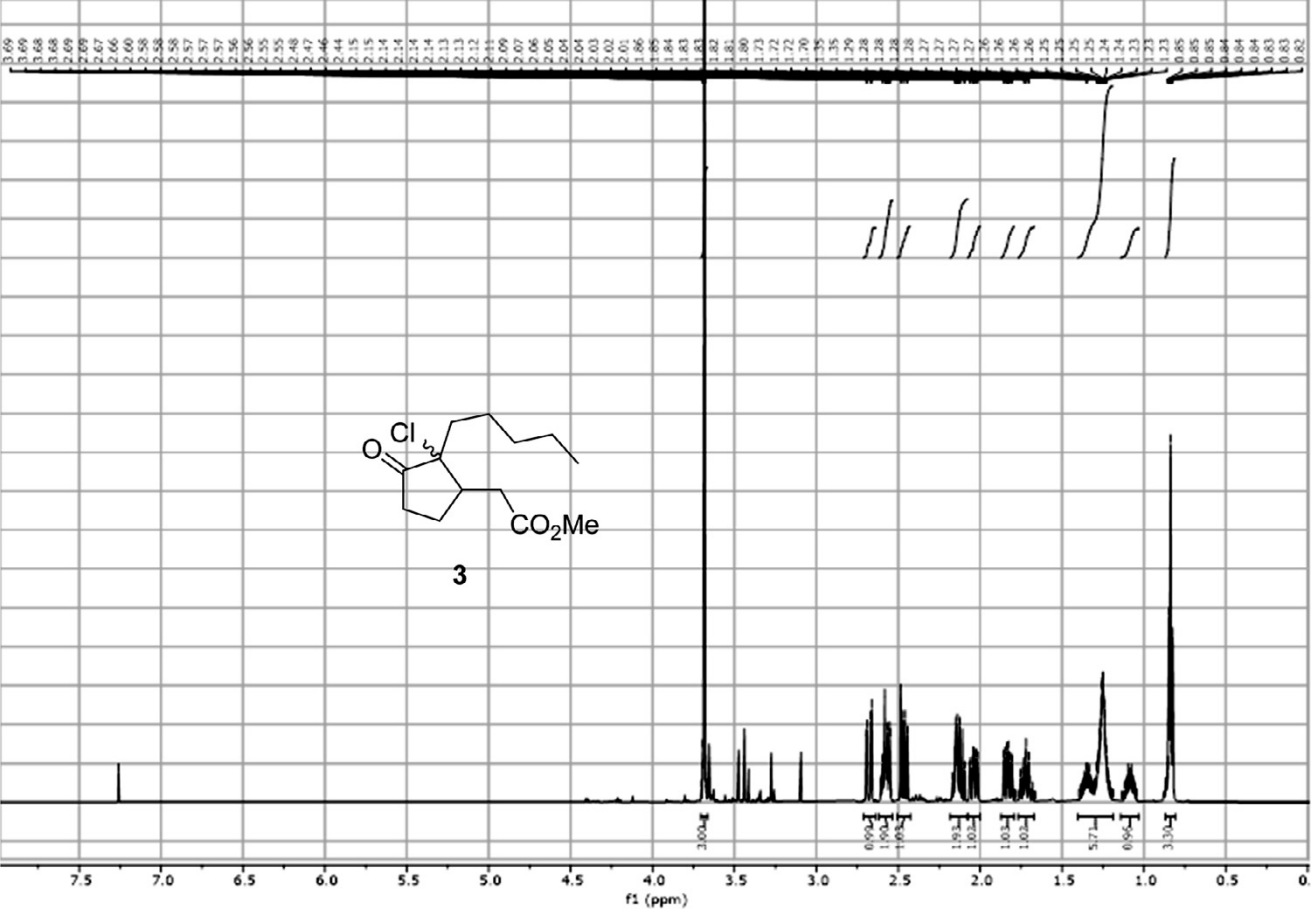
^1^H-NMR Spectra for the compound **3**[2].

**Fig. 15:**
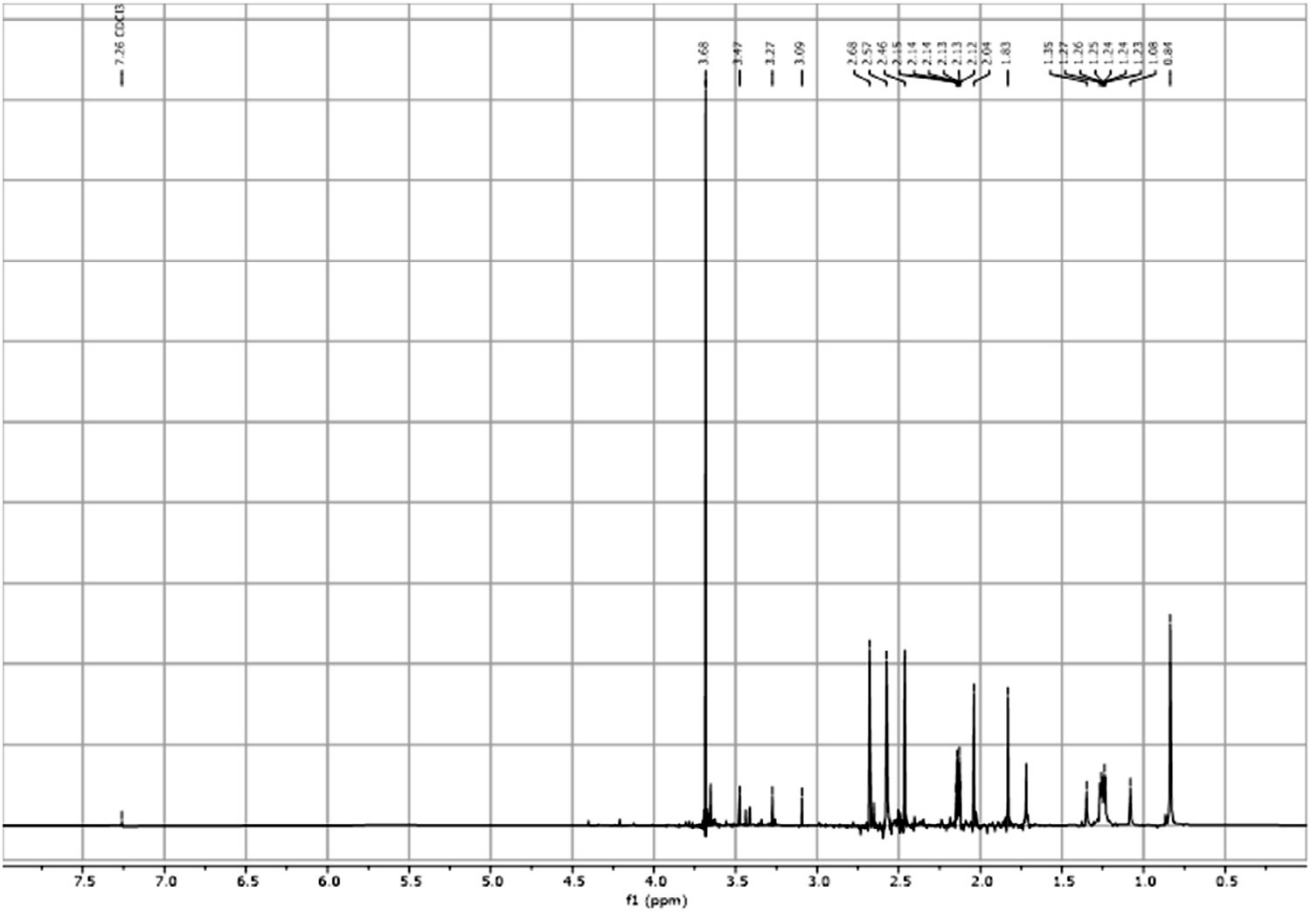
^1^H-PSYCHE spectra for the compound **3**[2].

**Fig. 16:**
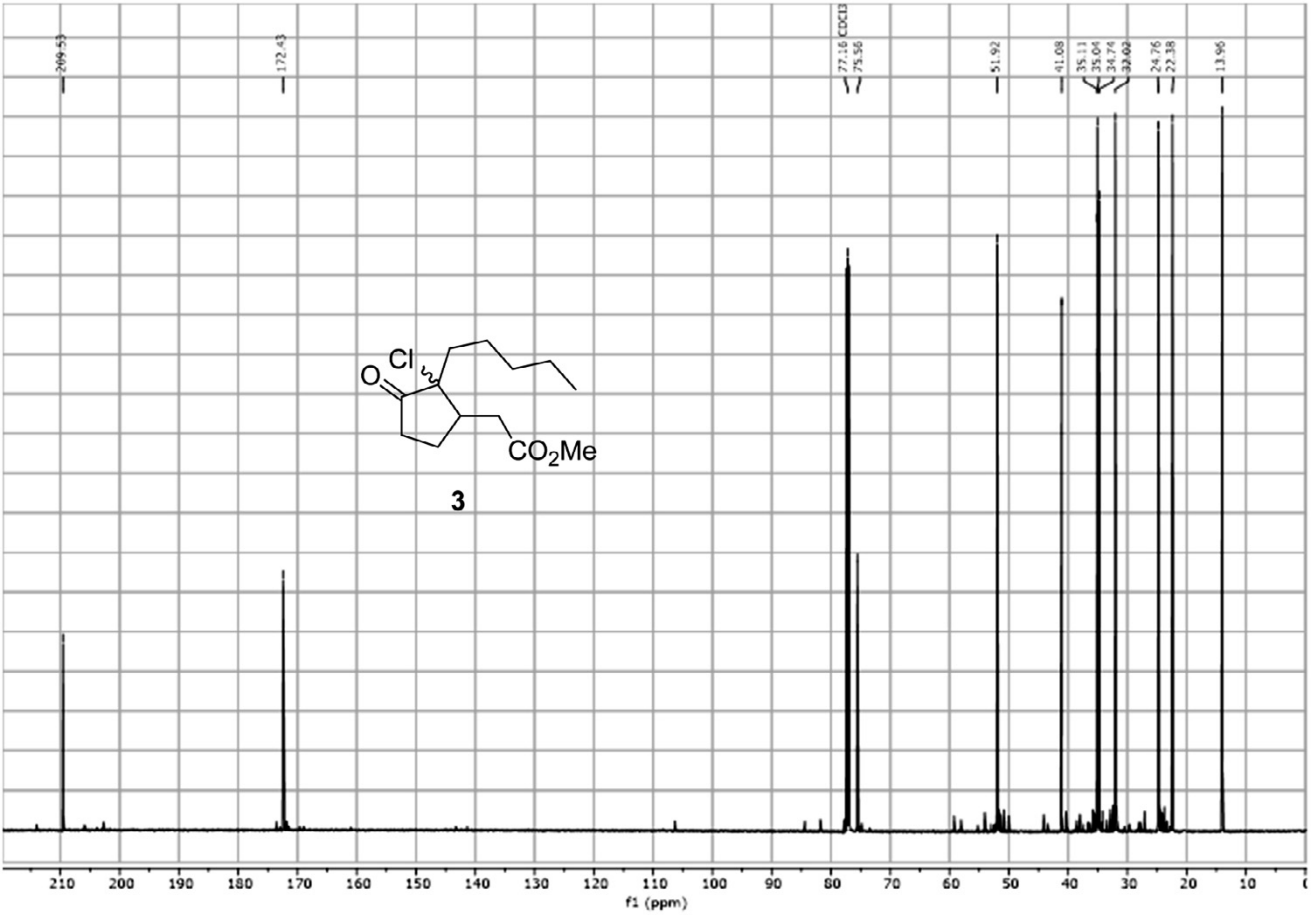
^13^C-NMR spectra for the compound **3**[2].

**Fig. 17:**
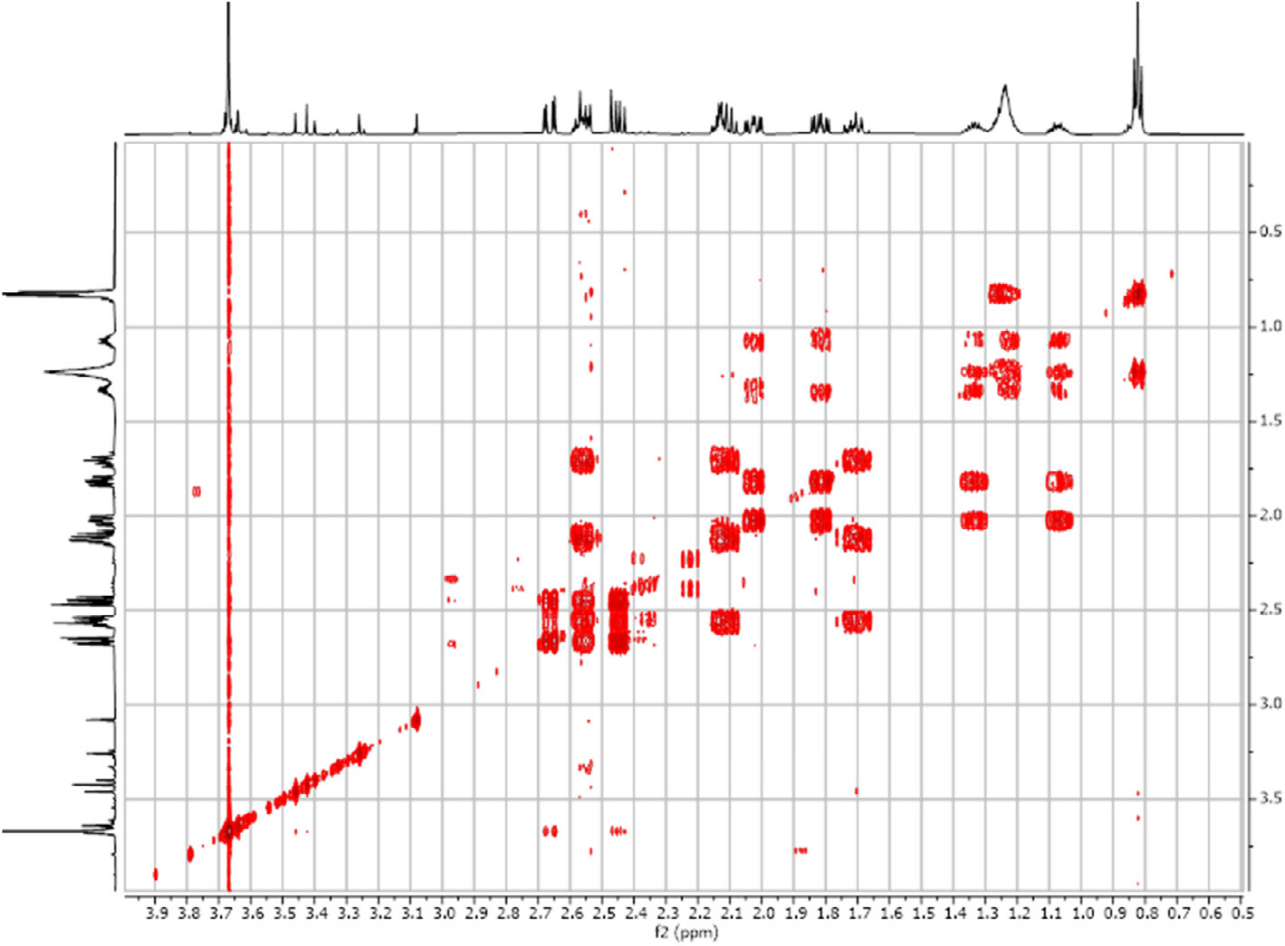
2-D ^1^H-^1^H COSY spectra for the compound **3**.

**Fig. 18:**
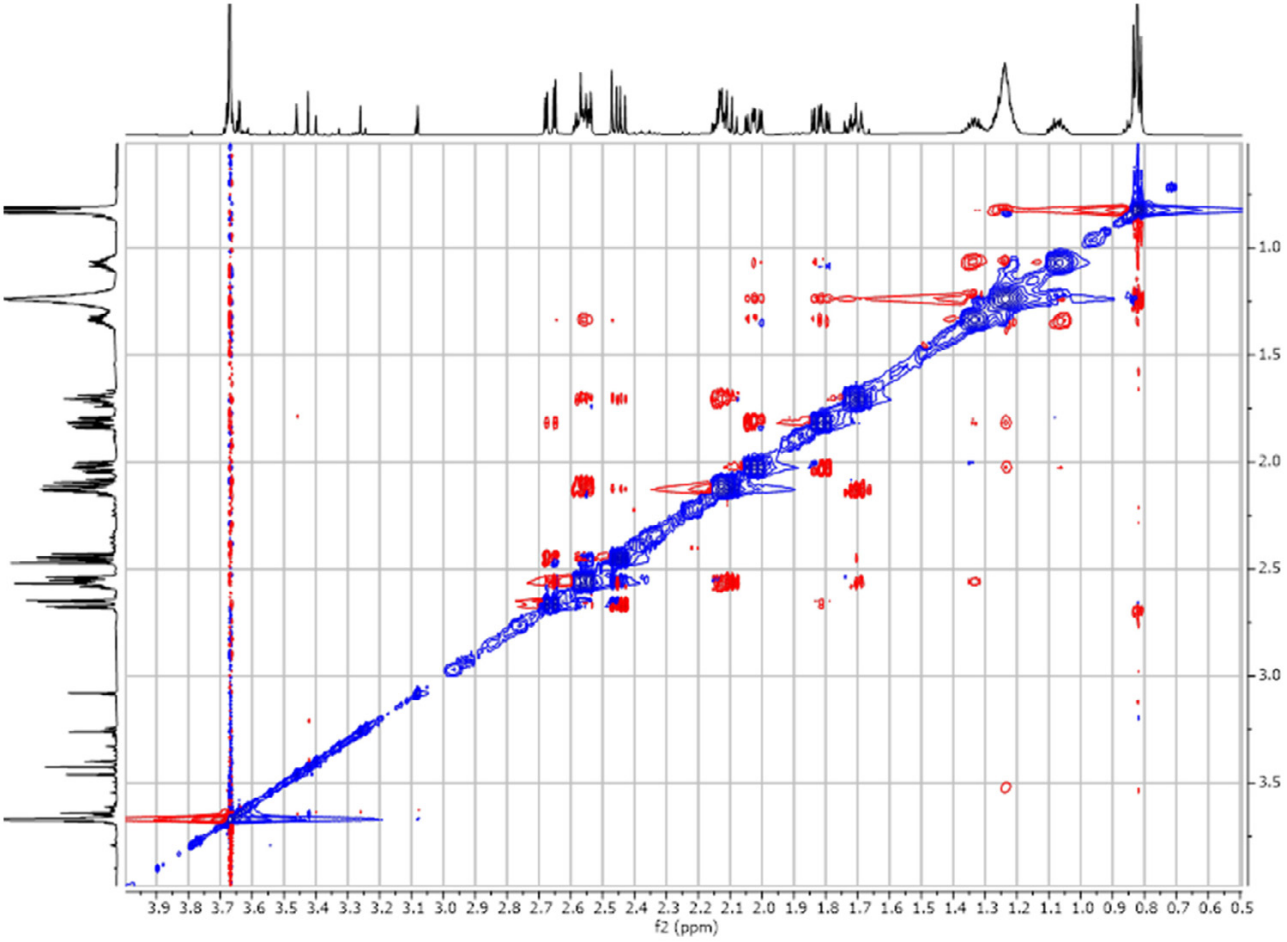
2-D ^1^H-^1^H NOESY spectra for the compound **3**.

**Fig. 19:**
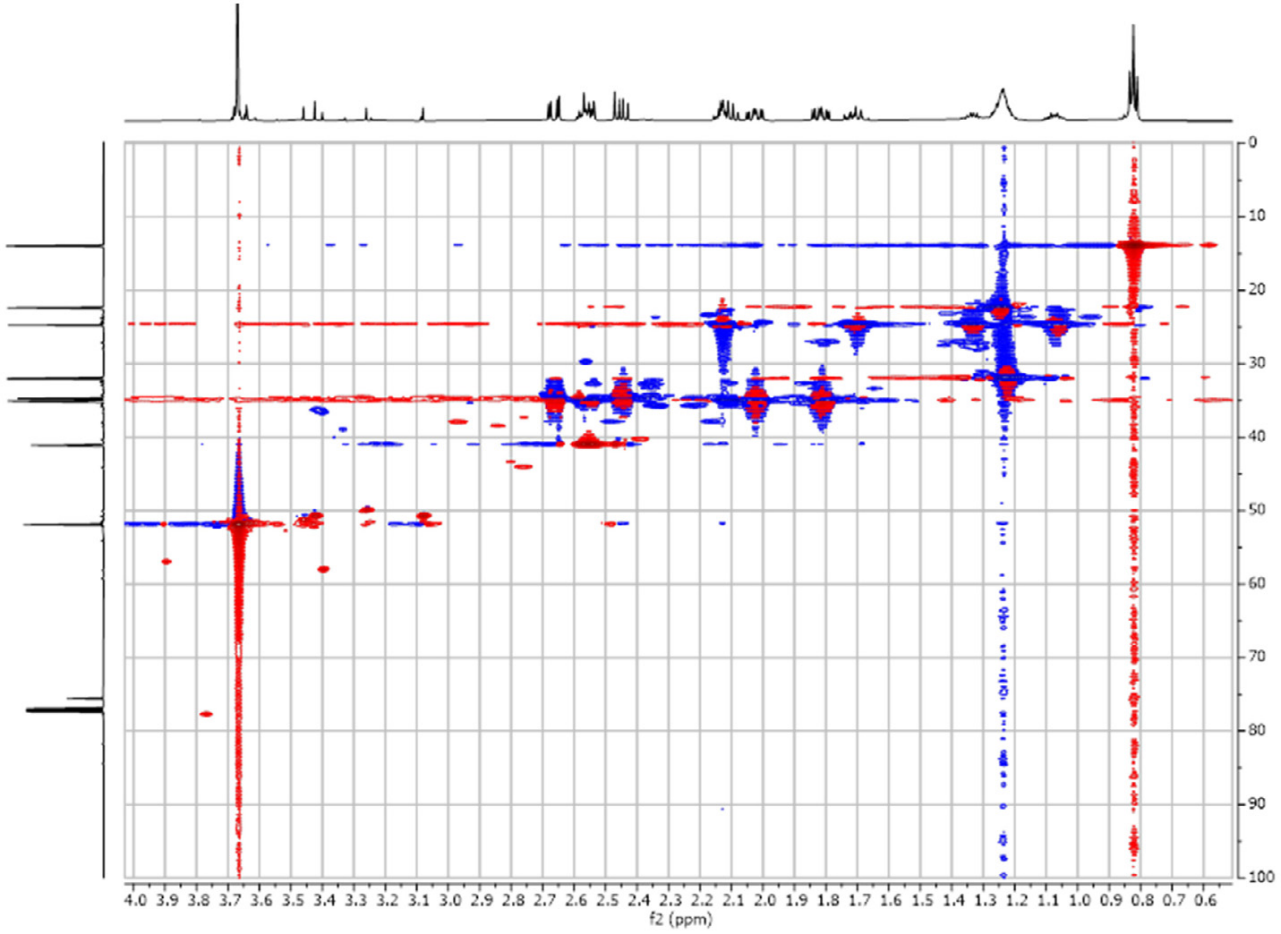
2-D ^1^H-^13^C HSQC spectra for the compound **3**.

**Fig. 20:**
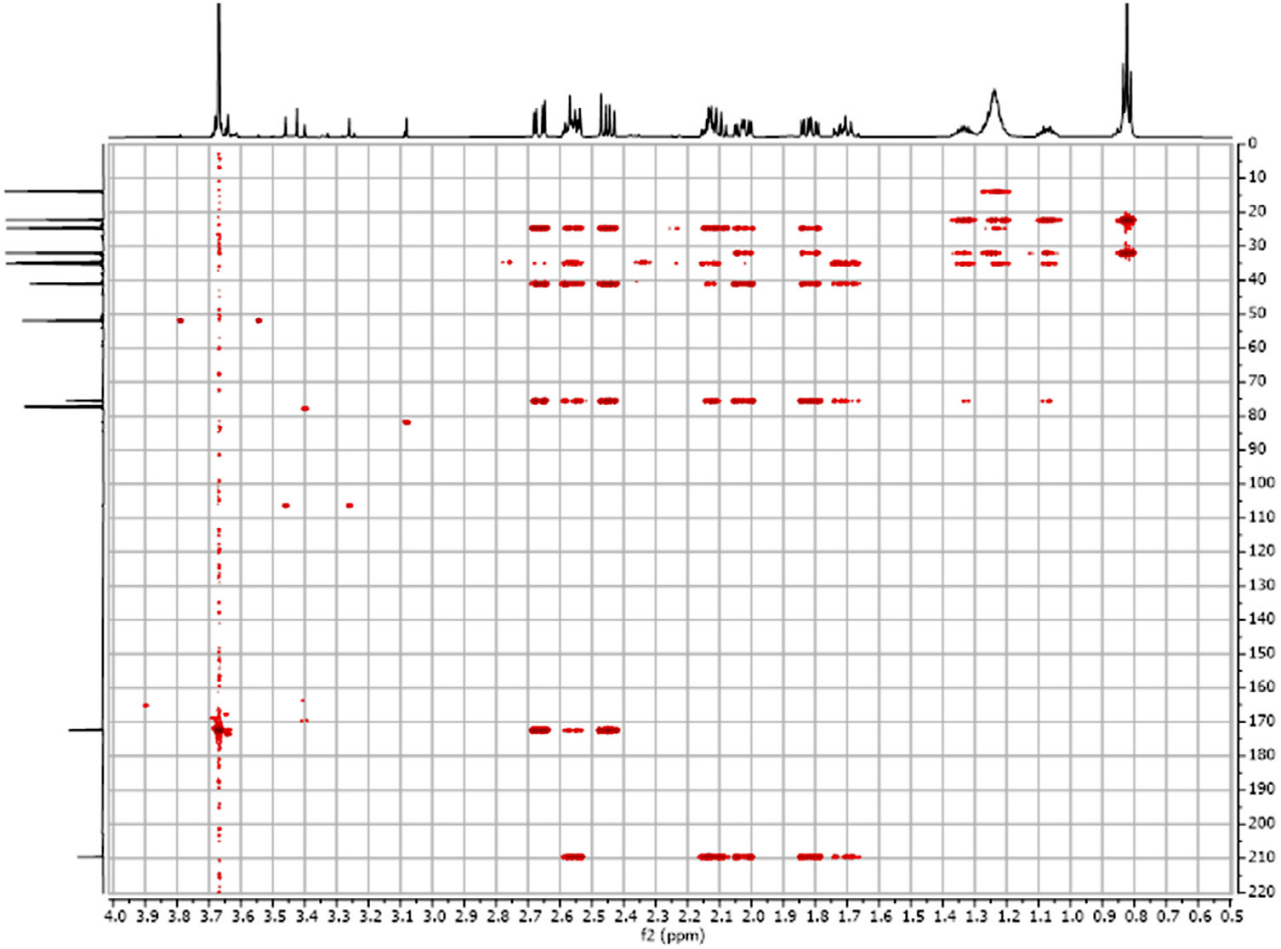
2-D ^1^H-^13^C HMBC spectra for the compound **3**.

**Fig. 21:**
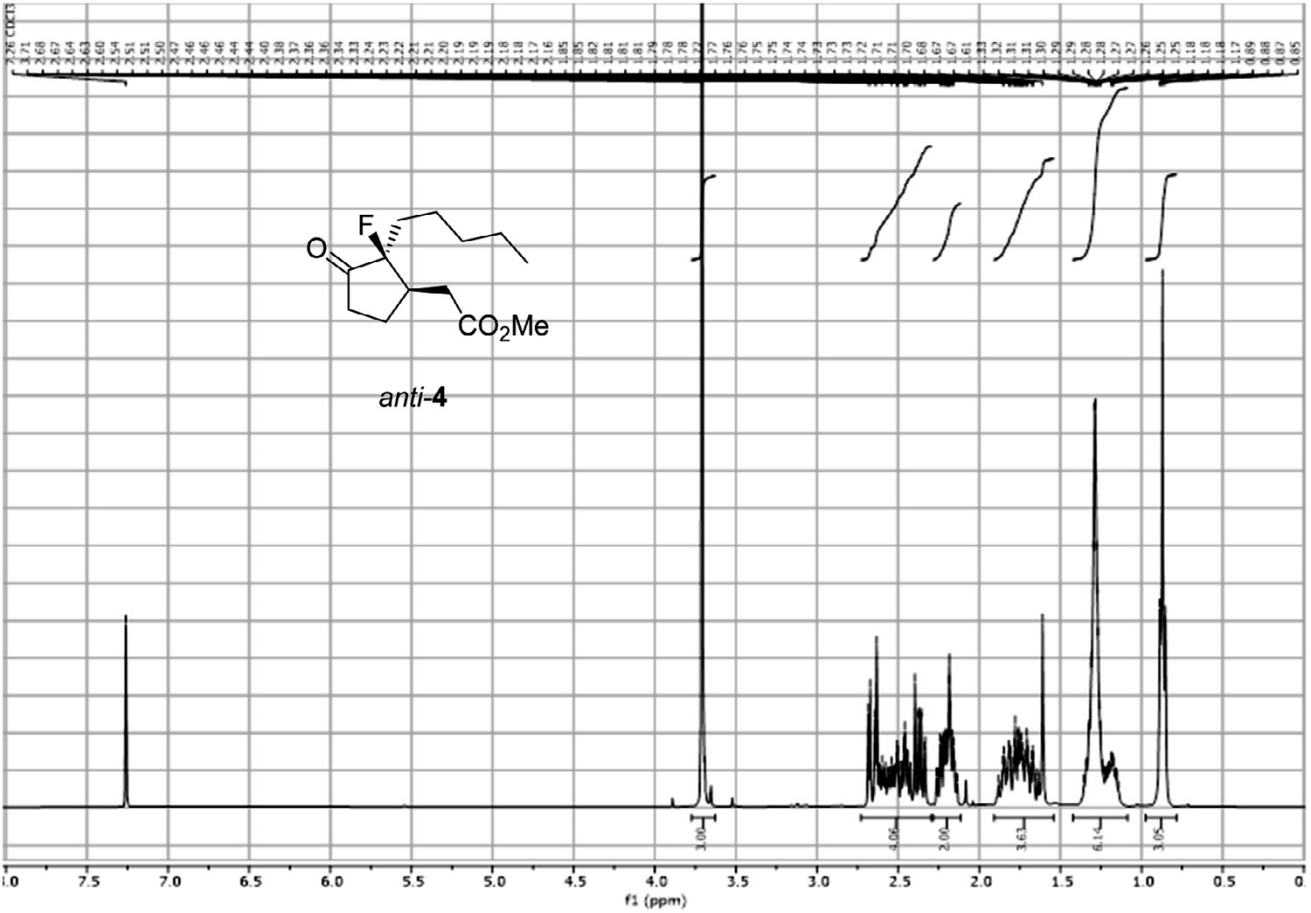
^1^H-NMR spectra for the compound *anti-***4**[2].

**Fig. 22:**
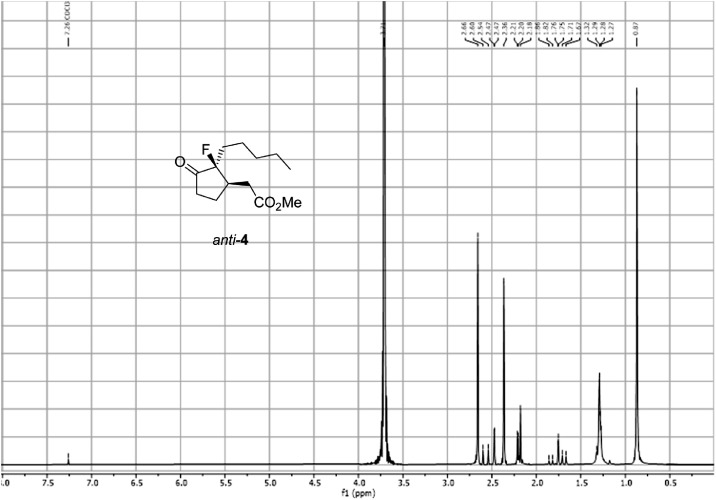
^1^H PSYCHE spectra for the compound *anti-***4**[2].

**Fig. 23:**
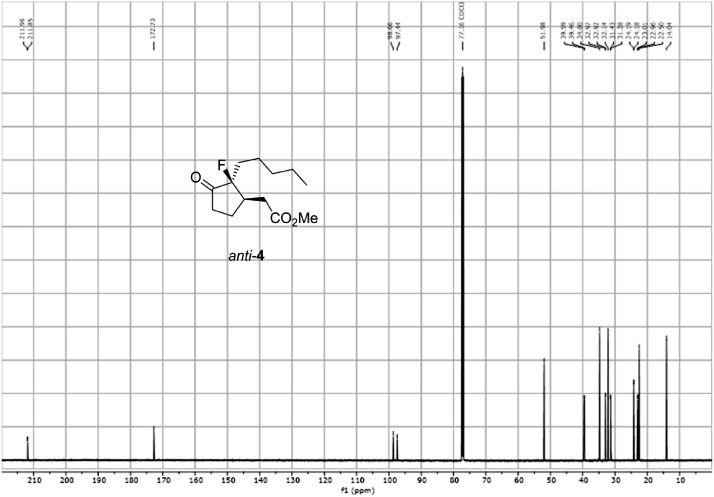
^13^C-NMR spectra for the compound *anti-***4**. [2]

**Fig. 24:**
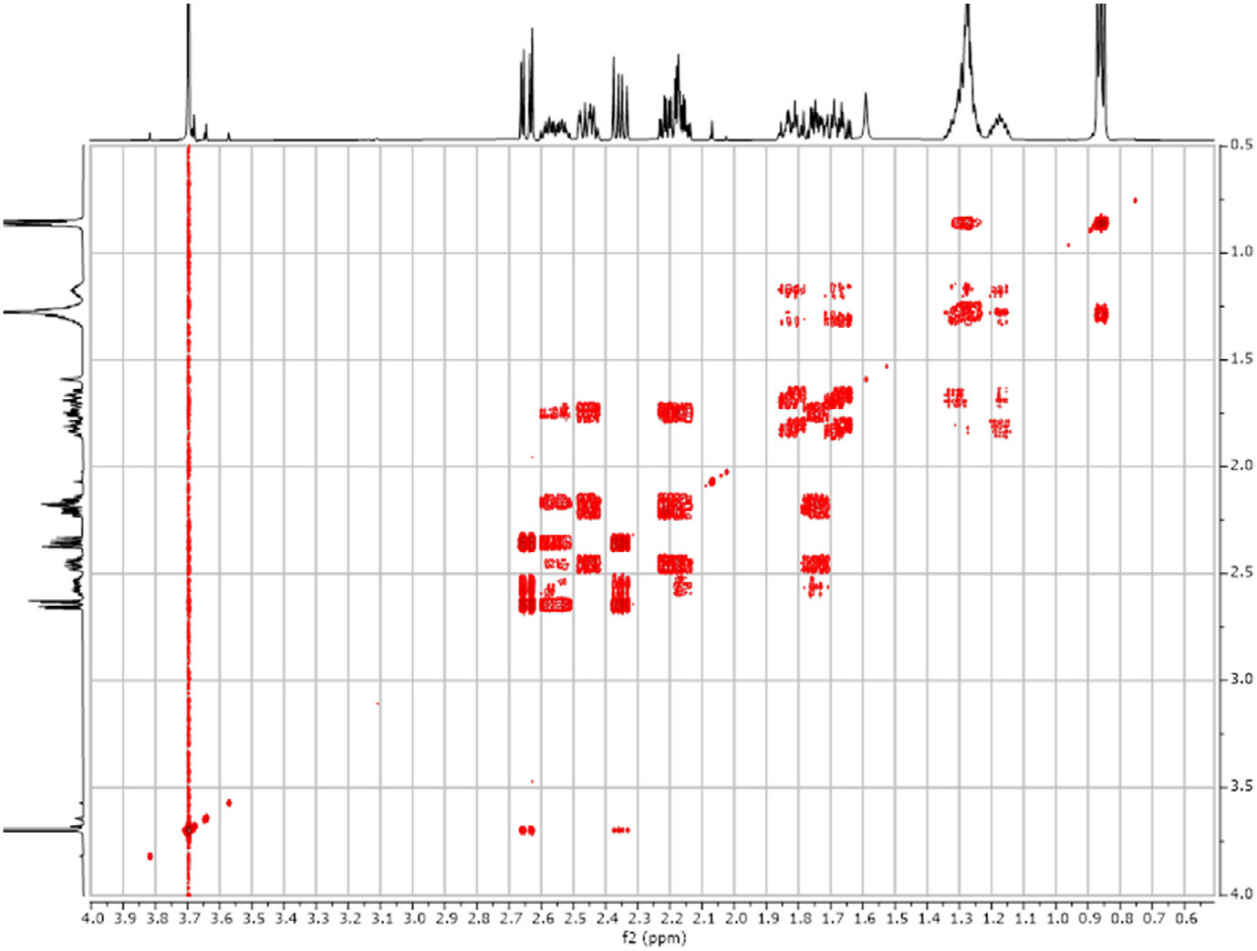
2-D ^1^H-^1^H COSY spectra for the compound *anti-***4**.

**Fig. 25:**
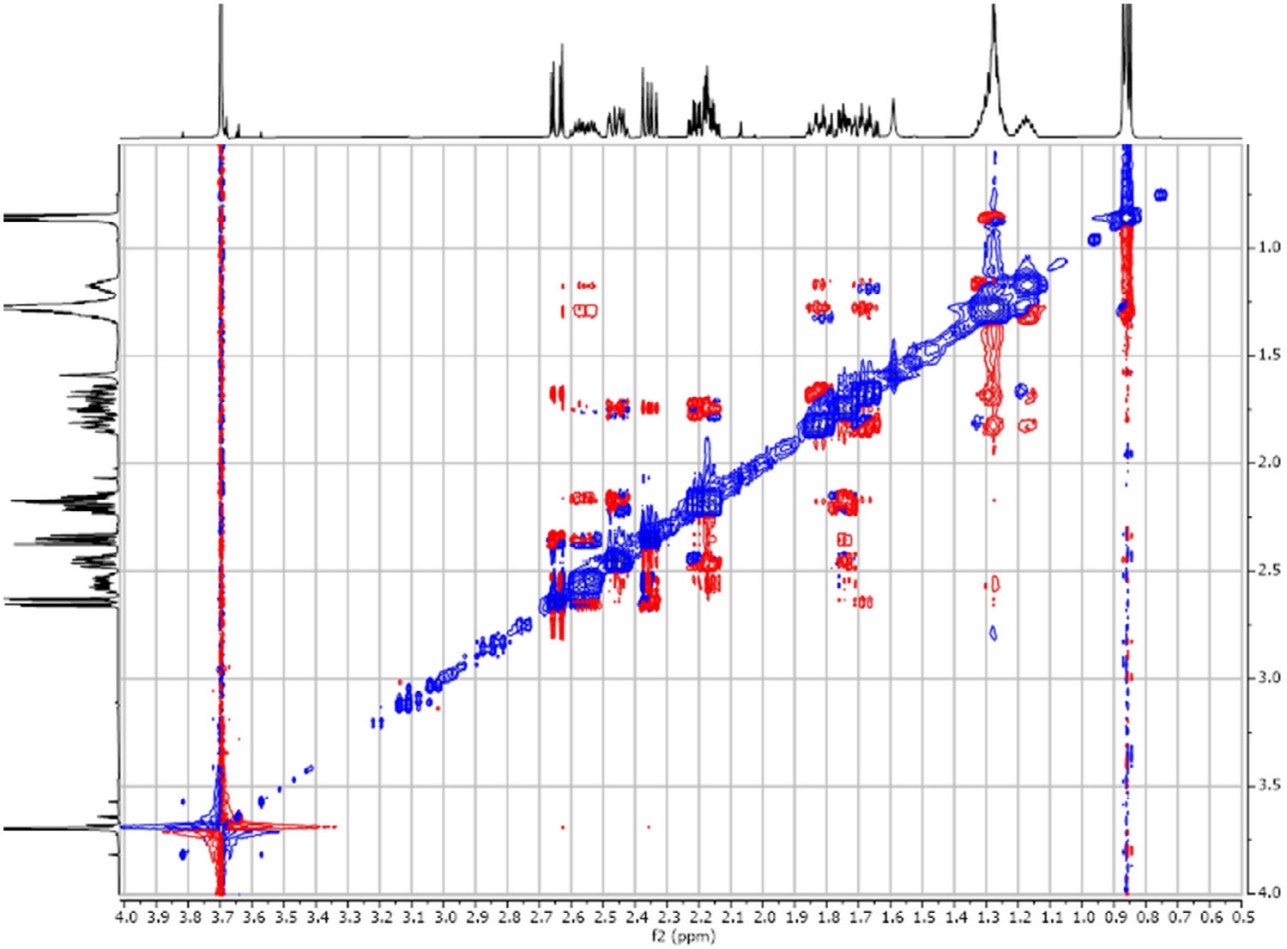
2-D ^1^H-^1^H NOESY spectra for the compound *anti-***4**.

**Fig. 26:**
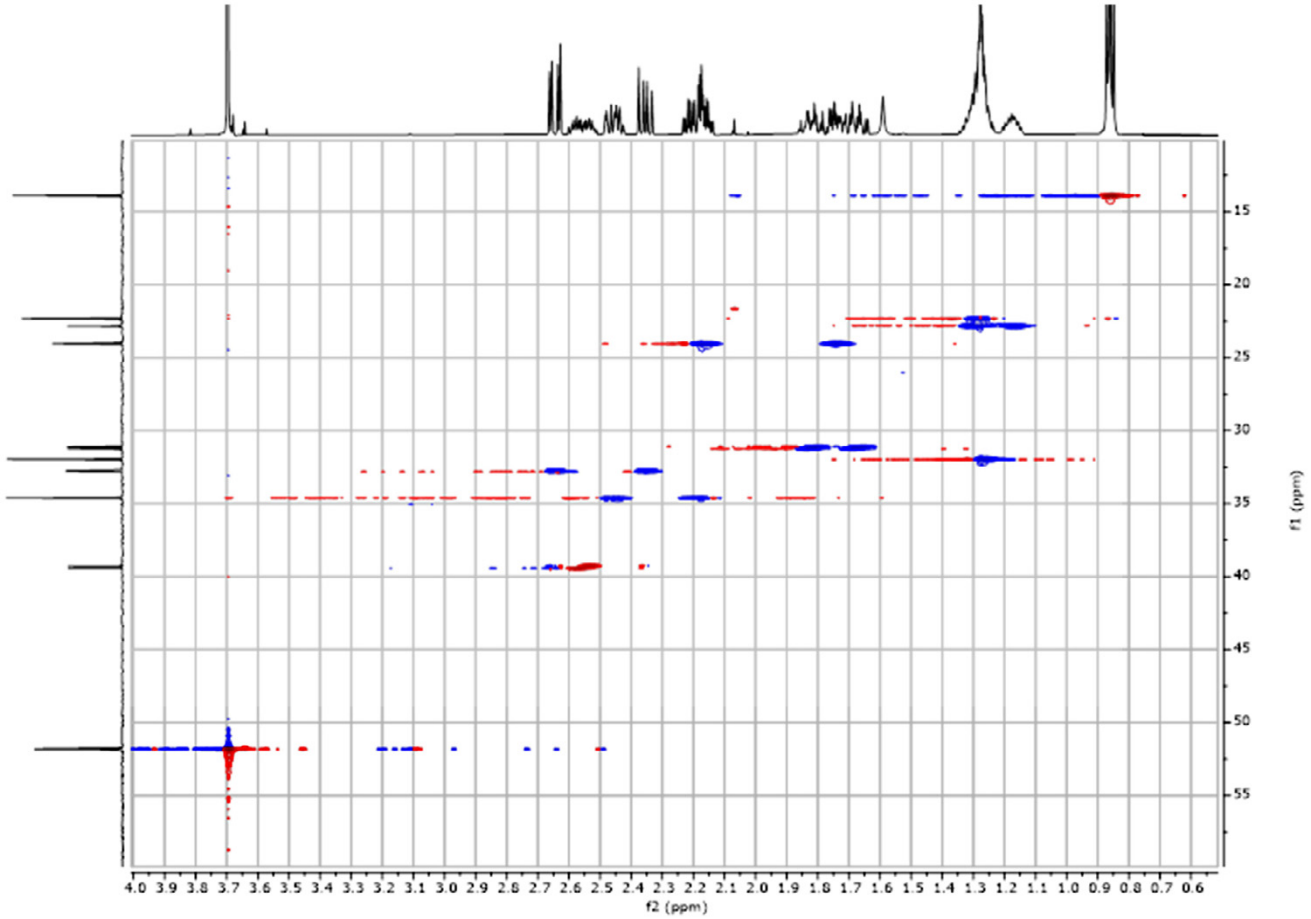
2-D ^1^H-^13^C HSQC spectra for the compound *anti-***4**.

**Fig. 27:**
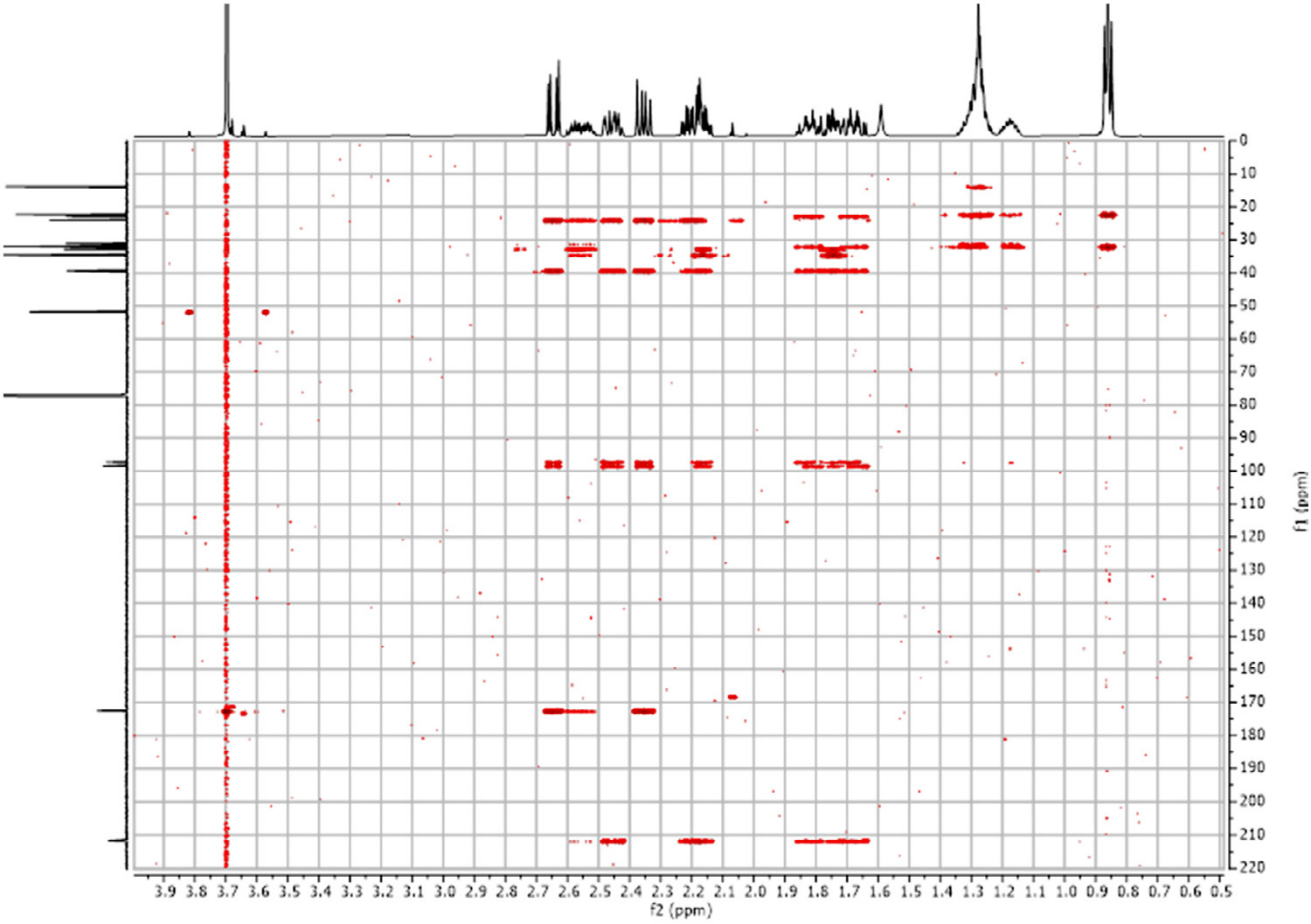
2-D ^1^H-^13^C HMBC spectra for the compound *anti-***4**.

**Fig. 28:**
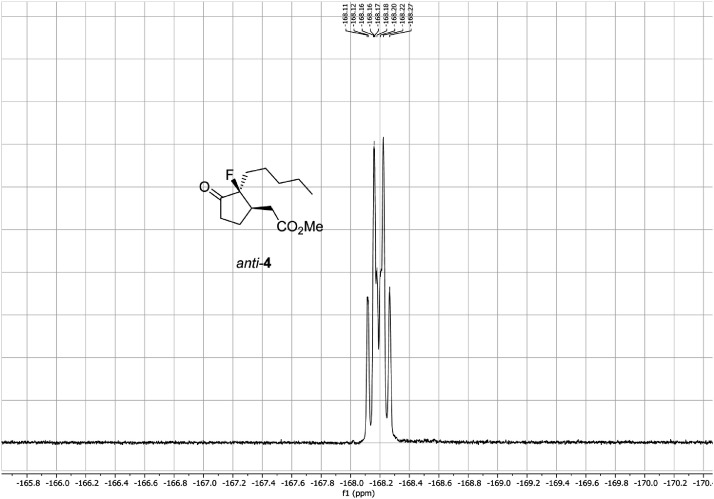
^19^F-NMR ^1^H-coupled spectra for the compound *anti-***4**[2].

**Fig. 29:**
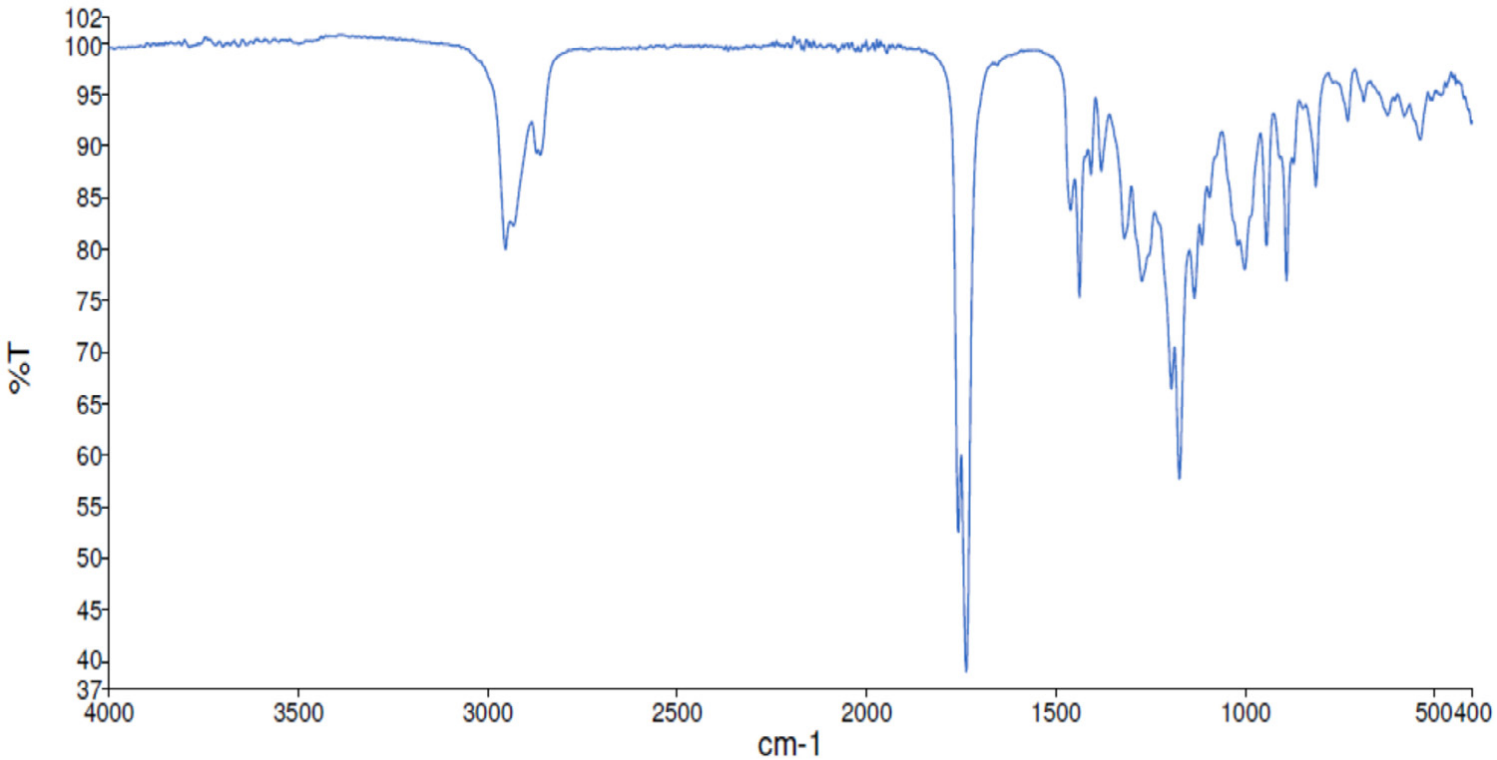
FT-IR spectra for the compound *anti-***4**[2].

**Fig. 30:**
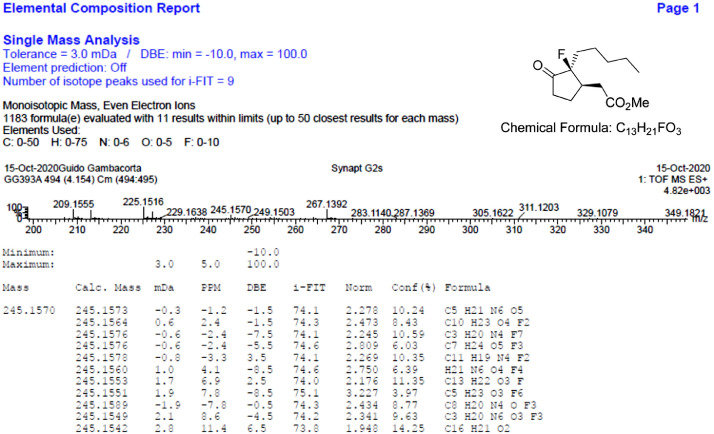
HR-MS report acquired for the compound *anti-***4**[2].

**Fig. 31:**
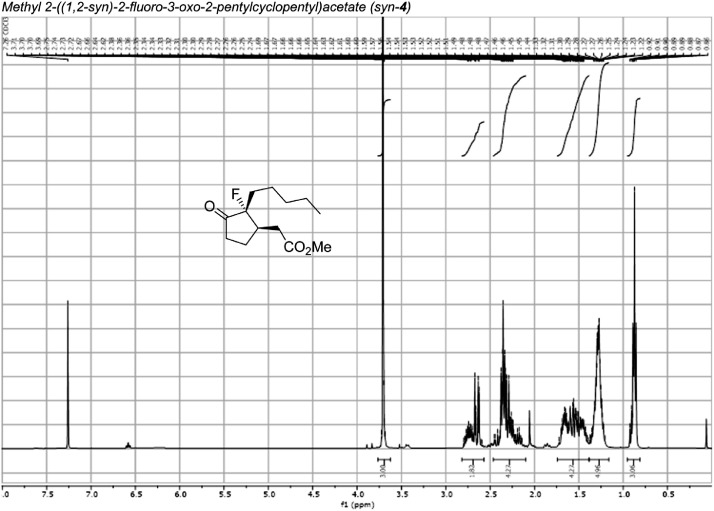
^1^H-NMR spectra for the compound *syn-***4**[2].

**Fig. 32:**
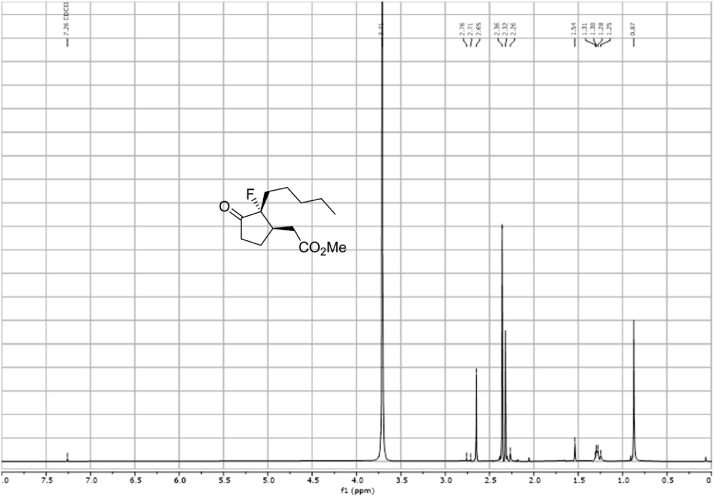
^1^H-PSYCHE spectra for the compound *syn-***4**[2].

**Fig. 33:**
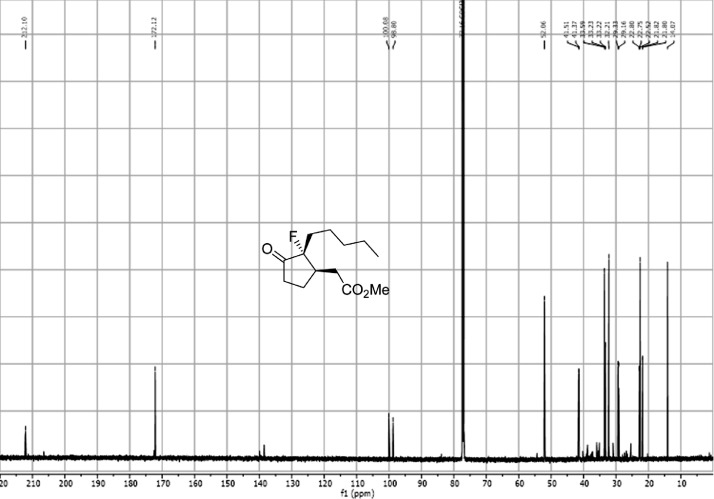
^13^C-NMR spectra for the compound *syn-***4**[2].

**Fig. 34:**
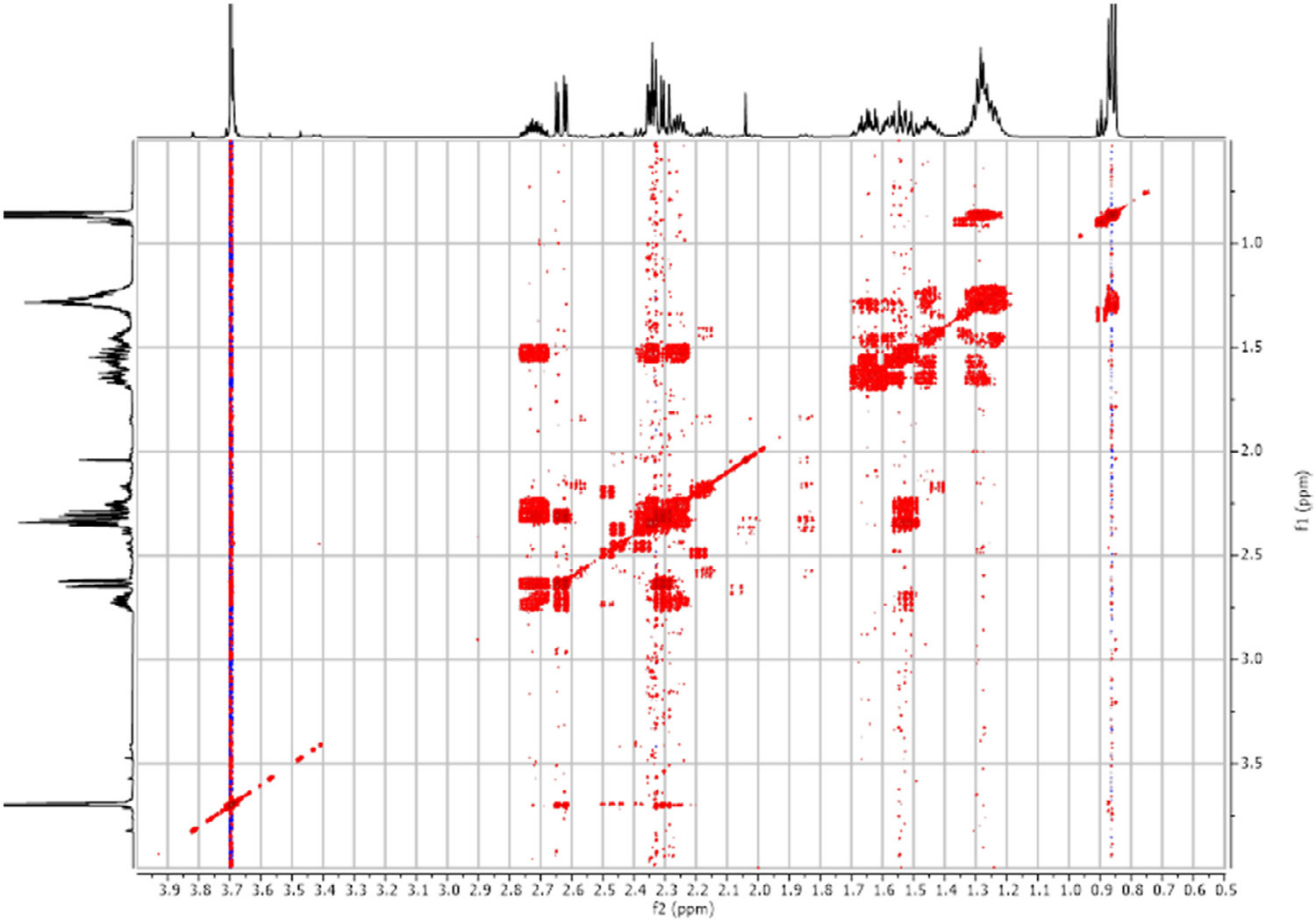
2-D ^1^H-^1^H COSY spectra for the compound *syn-***4**.

**Fig. 35:**
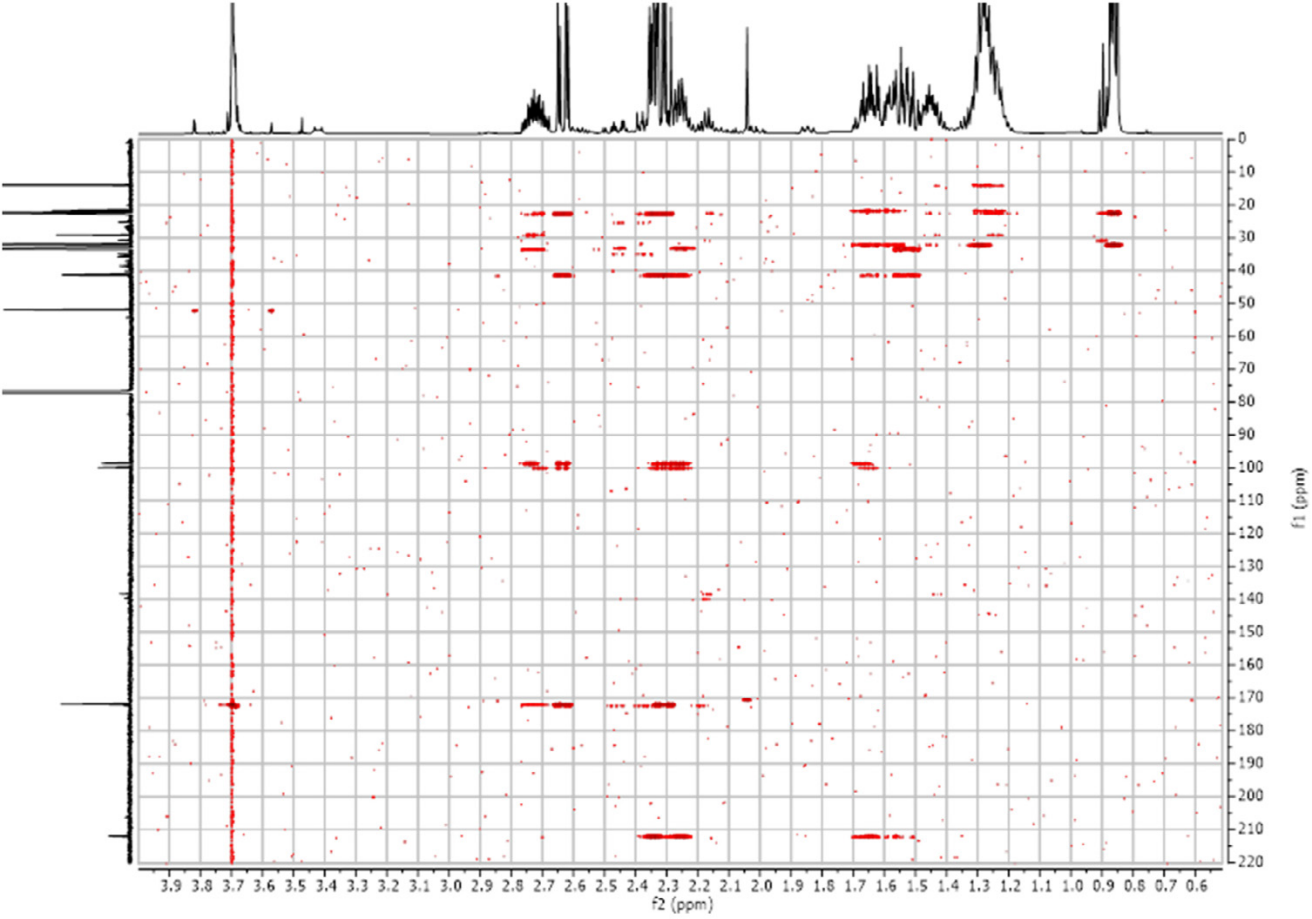
2-D ^1^H-^13^C HMBC spectra for the compound *syn-***4**.

**Fig. 36:**
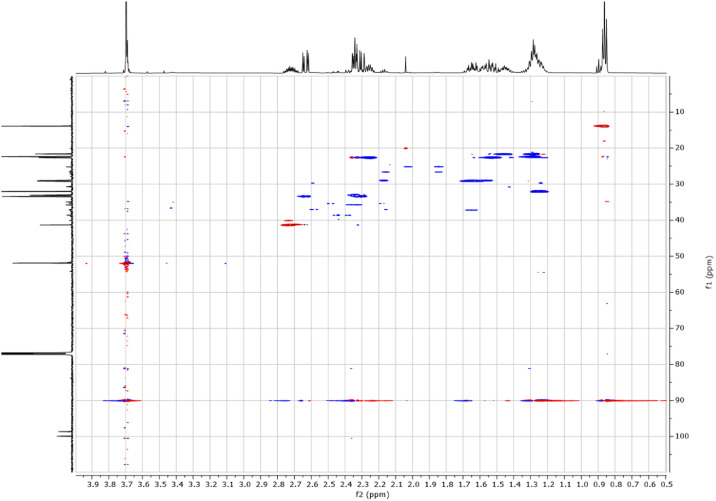
2-D ^1^H-^13^C HSQC spectra for the compound *syn-***4**.

**Fig. 37:**
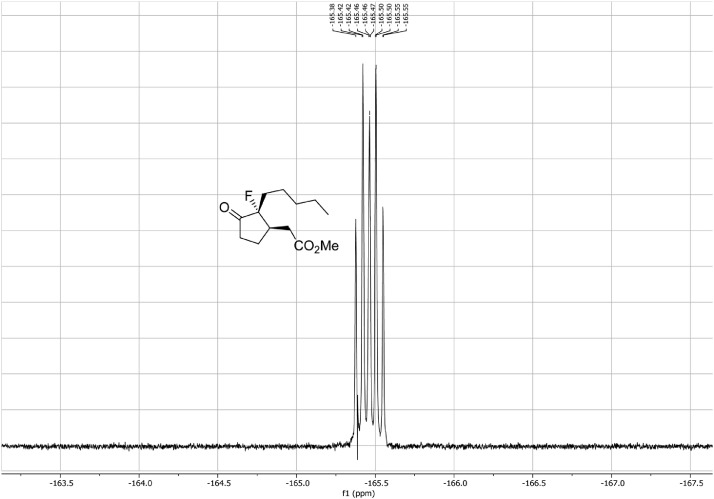
^19^F-NMR spectra for the compound *syn-***4**[2].

**Fig. 38:**
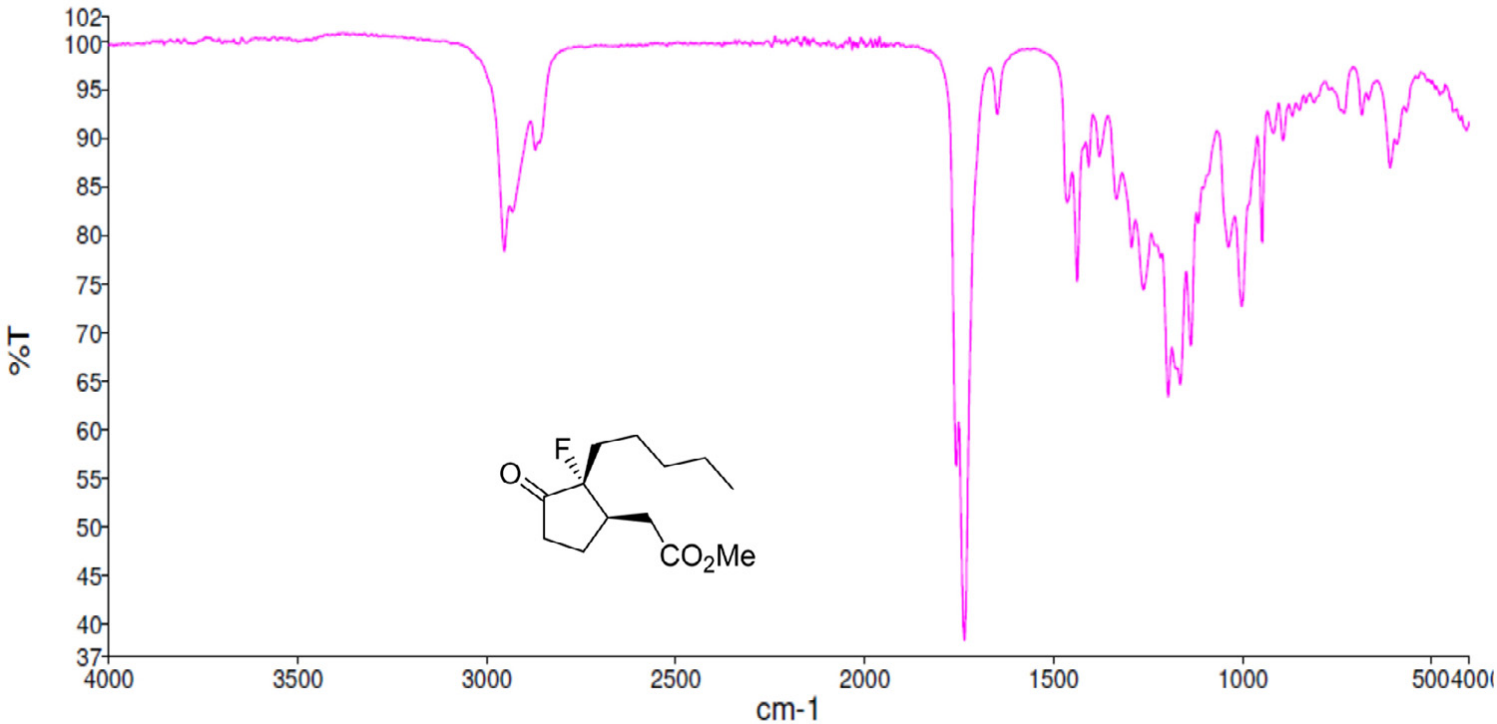
FT-IR spectra for the compound *syn-***4**[2].

**Fig. 39:**
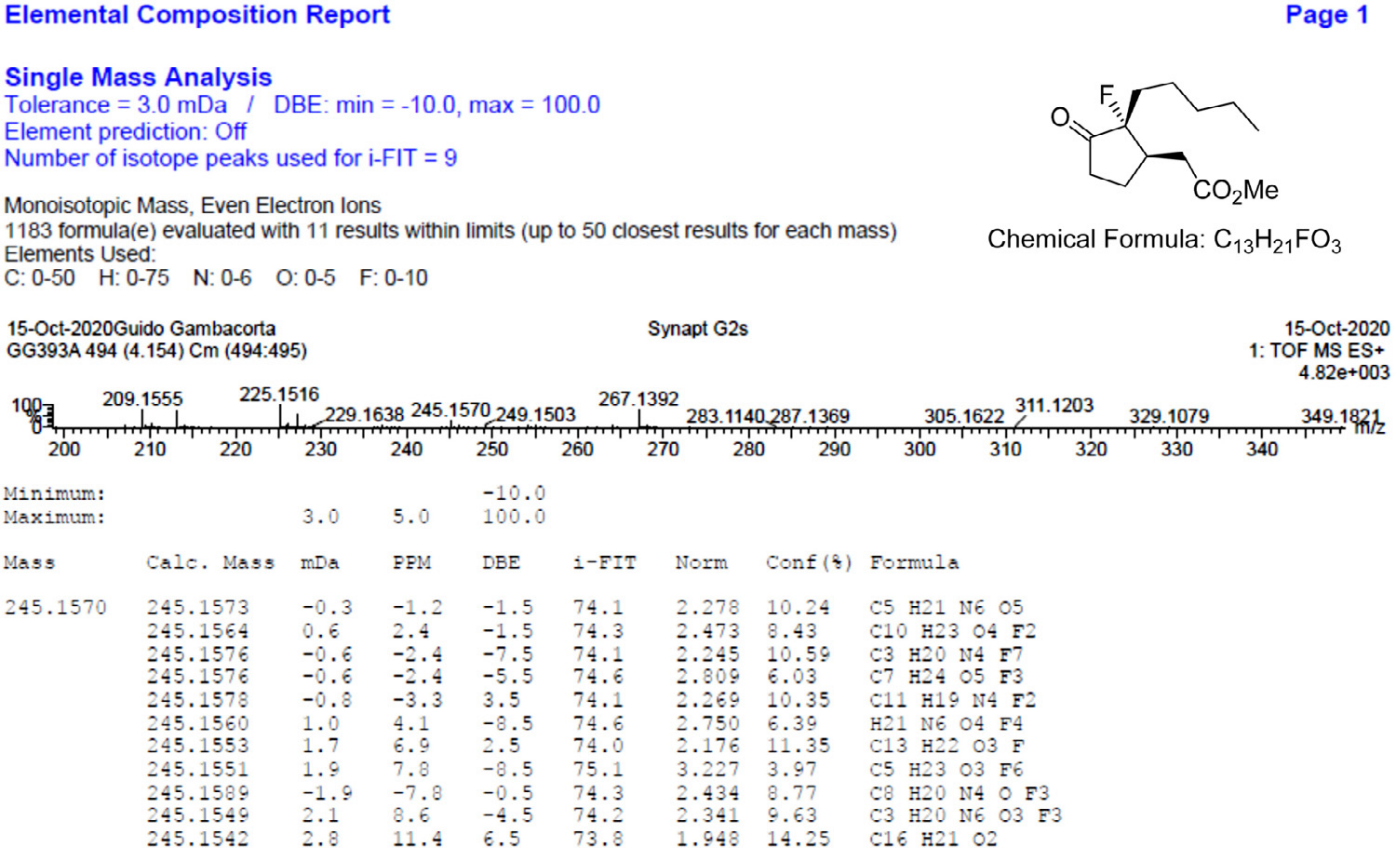
HR-MS report for the compound *syn-***4**[2].

## 2 Data Description

**Scheme 1:**
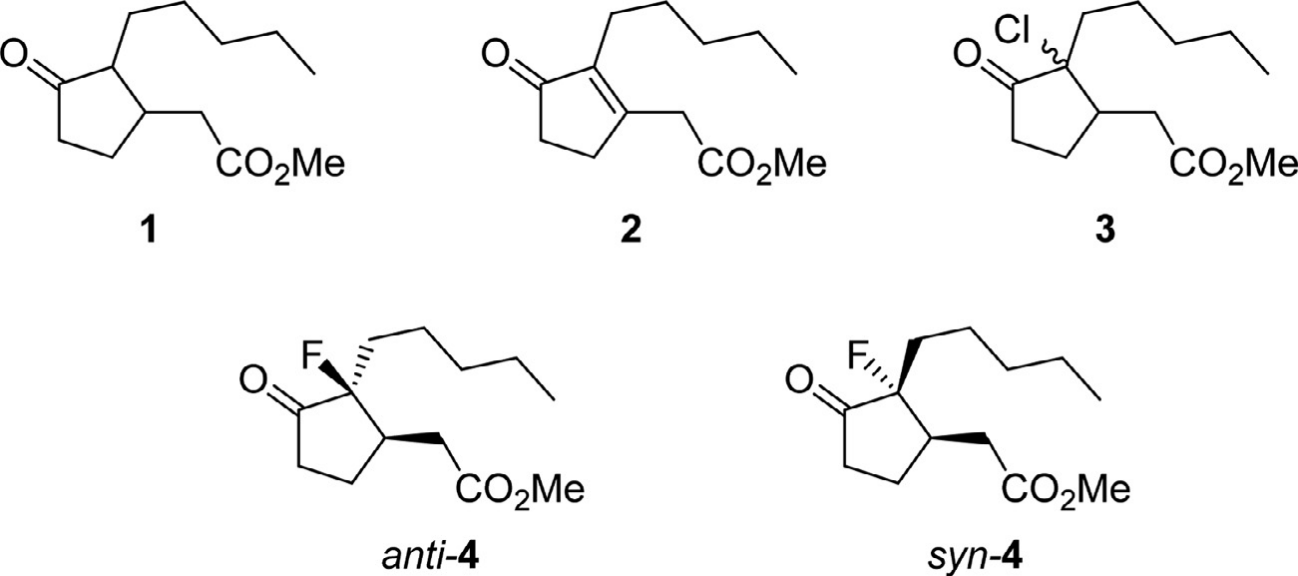
Summary representation of the molecules reported in the manuscript [2].

The data are divided into 5 sub-chapters as per the molecular structures identified during experimentation and as depicted in Scheme 1. Each sub-chapter contains the Nuclear magnetic resonance (NMR), Fourier-transform infrared spectroscopy (FT-IR), and high-resolution mass spectroscopy (HR-MS) reports acquired for the reported chemical compounds.

In respect to the NMR spectra depicted in the manuscript, the raw data were analyzed by means of Mestrenova v.14. The one-dimensional NMR Proton (^1^H) spectra represents the signals recorded for the specific purified molecule focused on the range between 0 and 8 ppm. The reference signal of the solvent (CDCl_3_ = 7.26 ppm) is highlighted in the spectra as well as the signals of the represented molecule. The Carbon-13 (^13^C) spectra shows the signals of the reported molecule focused on the range between 0 and 230 ppm. The reference signal of the solvent (CDCl_3_ = 77.16 ppm) is highlighted in the spectra as well as the signals representing the recorded molecule. For the reported molecules *anti-***4** and *syn-***4**, ^1^H-coupled Fluorine-19 (^19^F) NMR spectra and ^1^H-PSYCHE are also reported therein.

The dataset contains also two-dimensional NMR spectra (^1^H-^1^H COSY, ^1^H-^1^H NOESY, ^1^H-^13^C HSQC, ^1^H-^13^C HMBC) for each reported molecule **1**-**5**. The signals printed in red have a positive intensity (phase), whereas the signal printed in blue have a negative intensity (antiphase). These 2-D NMR spectra also depict the 1-D NMR spectra on the side to allow the reader to gain a better understanding of the data collected.

Along with the presented NMR, FT-IR spectra are also reported in the depository. The spectra correlate the grade of absorbance (in percentage transmittance, %T) that was acquired for each sample at the given wavelength in the range between 4000 and 400 cm^−1^.

The HR-MS reports are also reported for each identified molecule **1**-**5**. The data reported shows the mass spectrum recorded for a given molecule measured as a mass-to-charge ratio (m/z). The individual HR-MS reports present tabulated data of potential best fit structures giving a comparison between the measured mass (in figure named “Mass”) and the calculated masses (in figure named “Calc. Mass”). In this table, the difference between the measured mass and the calculated mass are also reported (as both mDa and ppm) as well as the maximum and minimum tolerance levels (as shown above the table). To facilitate the reading, the expected chemical formula is depicted in the top right-hand side of the figure. It is noteworthy, a hydrogen atom needs to be included in this chemical formula to be compared with the measured formula.

Raw data are publicly available on the Mendeley Data repository doi: 10.17632/2tfvzrmhzs.2 (https://data.mendeley.com/datasets/2tfvzrmhzs).

## 3 Experimental Design, Materials and Methods

All the chemical compounds presented herein described were either supplied by International Flavors & Fragrance Inc. (IFF) or isolated in the laboratory following procedures reported in the related article [2].

The Nuclear magnetic resonance spectra were acquired on either Bruker Avance-400 (^19^F-NMRs) or Varian VNMRS-600 (^1^H, ^13^C, and 2-D NMRs). Samples were prepared by solubilizing 20 mg of substance in 1 mL of CDCl_3_ of solvent and insert it into a NMR tube for acquisition. The NMR data acquisition was carried out by the NMR service provided by Durham University. The data were analyzed by means of Mestrenova version 14 under the license of Durham University.

FT-IR spectra were acquired employing a Perkin Elmer Spectrum Two UATR Two FT-IR Spectrometer where the neat sample was spread on the detector.

HR-MS reports were obtained employing a Waters QtoF Premier mass spectrometer operated by Mass spectroscopy service provided by Durham University.

## Ethics Statement

The work does not involve human subjects, animal experiments, or data collected from social media platforms.

## CRediT authorship contribution statement

**James S. Sharley:** Conceptualization, Methodology, Validation, Writing – original draft. **Guido Gambacorta:** Conceptualization, Methodology, Validation, Writing – original draft. **Ana María Collado Pérez:** Writing – review & editing. **Estela Espinos Ferri:** Writing – review & editing. **Amadeo Fernandez Miranda:** Writing – review & editing. **Jorge Sanchez Quesada:** Project administration. **Ian R. Baxendale:** Supervision.

## Declaration of Competing Interest

The authors declare that they have no known competing financial interests or personal relationships that could have appeared to influence the work reported in this paper.

## Data Availability

Characterisation of Hedione's derivative compounds (Original data) (Mendeley Data).
